# Temporal trends and patterns in initial opioid prescriptions after hospital discharge following colectomy in England over 10 years

**DOI:** 10.1093/bjsopen/zrad136

**Published:** 2023-12-26

**Authors:** Reham M Baamer, David J Humes, Li Shean Toh, Roger D Knaggs, Dileep N Lobo

**Affiliations:** Division of Pharmacy Practice and Policy, School of Pharmacy, University of Nottingham, Nottingham, UK; Department of Pharmacy Practice, Faculty of Pharmacy, King Abdulaziz University, Jeddah, Saudi Arabia; Nottingham Digestive Diseases Centre, Division of Translational Medical Sciences, School of Medicine, University of Nottingham, Queen’s Medical Centre, Nottingham, UK; National Institute for Health Research Nottingham Biomedical Research Centre, Nottingham University Hospitals NHS Trust and University of Nottingham, Queen’s Medical Centre, Nottingham, UK; Division of Pharmacy Practice and Policy, School of Pharmacy, University of Nottingham, Nottingham, UK; Division of Pharmacy Practice and Policy, School of Pharmacy, University of Nottingham, Nottingham, UK; Pain Centre Versus Arthritis, University of Nottingham, Nottingham, UK; Nottingham Digestive Diseases Centre, Division of Translational Medical Sciences, School of Medicine, University of Nottingham, Queen’s Medical Centre, Nottingham, UK; National Institute for Health Research Nottingham Biomedical Research Centre, Nottingham University Hospitals NHS Trust and University of Nottingham, Queen’s Medical Centre, Nottingham, UK; David Greenfield Metabolic Physiology Unit, MRC Versus Arthritis Centre for Musculoskeletal Ageing Research, School of Life Sciences, University of Nottingham, Queen’s Medical Centre, Nottingham, UK; Department of Surgery, Perelman School of Medicine, University of Pennsylvania, Philadelphia, Pennsylvania, USA

## Abstract

**Background:**

While opioid analgesics are often necessary for the management of acute postoperative pain, appropriate prescribing practices are crucial to avoid harm. The aim was to investigate the changes in the proportion of people receiving initial opioid prescriptions after hospital discharge following colectomy, and describe trends and patterns in prescription characteristics.

**Methods:**

This was a retrospective cohort study. Patients undergoing colectomy in England between 2010 and 2019 were included using electronic health record data from linked primary (Clinical Practice Research Datalink Aurum) and secondary (Hospital Episode Statistics) care. The proportion of patients having an initial opioid prescription issued in primary care within 90 days of hospital discharge was calculated. Prescription characteristics of opioid type and formulation were described.

**Results:**

Of 95 155 individuals undergoing colectomy, 15 503 (16.3%) received opioid prescriptions. There was a downward trend in the proportion of patients with no prior opioid exposure (opioid naive) who had a postdischarge opioid prescription (*P* <0.001), from 11.4% in 2010 to 6.7% in 2019 (−41.3%, *P* <0.001), whereas the proportions remained stable for those prescribed opioids prior to surgery, from 57.5% in 2010 to 58.3% in 2019 (*P* = 0.637). Codeine represented 44.5% of all prescriptions and prescribing increased by 14.5% between 2010 and 2019. Prescriptions for morphine and oxycodone rose significantly by 76.6% and 31.0% respectively, while tramadol prescribing dropped by 48.0%. The most commonly prescribed opioid formulations were immediate release (83.9%), followed by modified release (5.8%) and transdermal (3.2%). There was a modest decrease in the prescribing of immediate-release formulations from 86.0% in 2010 to 82.0% in 2019 (*P* <0.001).

**Conclusion:**

Over the 10 years studied, there was a changing pattern of opioid prescribing following colectomy, with a decrease in the proportion of opioid-naive patients prescribed postdischarge opioids.

## Teaser

This retrospective cohort study investigated the changes in the proportion of people receiving initial opioid prescriptions after hospital discharge following colectomy, and describes trends and patterns in prescription characteristics. It found that there was a changing pattern of opioid prescribing following colectomy, with a decrease in the proportion of opioid-naive patients prescribed postdischarge opioids.

## Introduction

While opioid analgesics are often necessary for the management of acute postoperative pain, appropriate prescribing practices are crucial to avoid harm^1–3^. Postoperative opioid prescribing may lead to persistent postoperative opioid use (PPOU)^4,5^, with dependence differing by opioid type and likeability^6,7^. The authors' previous work demonstrated that 2.5% of opioid-naive patients who underwent colectomy in England developed PPOU, increasing to 40.4% in those with current opioid exposure^8^. Although definitions of PPOU vary^9,10^, in North America the incidence of PPOU can range from 0.6% to 12% in opioid-naive patients following abdominopelvic surgery and can be higher in those with previous opioid exposure^9^.

Differences in healthcare systems might have an impact on opioid prescribing practices. The UK has a publicly funded National Health Service (NHS), which provides free healthcare to all residents, including subsidized prescriptions^11^, mainly guided by national policies and drug formularies. In comparison, the USA has a private healthcare system linked predominantly to insurance coverage. The variation in patterns of postoperative opioid use can also be linked to the promotion of certain opioids over others, leading to differences in the opioid selection, duration and quantity prescribed to each patient, and between countries^12–14^. For instance, the rate of filled opioid prescriptions in the USA and Canada in the first week after discharge was seven times higher than in Sweden^4^. Although the frequency of filled prescriptions was similar between Canada and the USA, patients in US hospitals received greater quantities of opioids^4^. Additionally, from 1994 to 2014, 80% of patients undergoing surgery, including colectomy, in the US received postdischarge opioid prescriptions^5^.

Opioid-related adverse events, which are related to potency, formulation and dose, can be reduced by avoiding long-acting and transdermal formulations^2,15,16^. Opioids should only be prescribed as immediate-release formulations for management of postoperative pain^2,15^. Nevertheless, a recent UK study revealed that 10% of previously opioid-naive patients were discharged with long-acting formulations^17^. Additionally, in the USA, patients who were prescribed long-acting opioid formulations following surgical discharge were more likely to obtain prescriptions with higher doses compared with short-acting formulations. Prescriptions with long-acting formulations resulted in a total oral morphine equivalent (OMEQ) dose exceeding 350 mg^5^.

While evidence suggests a recent decline in postoperative opioid prescriptions in the USA^18,19^, it is unclear whether a similar trend exists in England. In addition, a comprehensive understanding of the specific opioids and formulations prescribed after surgical discharge and their variation over the years remains unexplored. This study aimed to investigate the changes in the proportion of people receiving initial opioid prescriptions after hospital discharge following colectomy, as well as describe trends and patterns in prescription characteristics, particularly temporal changes in analgesics, including potency and formulation choices.

## Methods

This retrospective cohort study was approved by the Independent Scientific Advisory Committee approval board (Protocol 21_000668) and followed the Reporting of Studies Conducted using Observational Routinely Collected Health Data Statement for Pharmacoepidemiology (RECORD-PE) guidelines^20^.

A repeated cross-sectional analysis was used to describe temporal trends of patients undergoing colectomy and prescribed opioids within 90 days of hospital discharge following surgery^8^, split by year-by-year data to describe trends and changes from 2010 to 2019. The 90-day duration was chosen to ensure that the prescribed opioid was associated with a surgical procedure, considering that the tissue healing process can take up to 3 months^21^. Moreover, this 90-day interval aligns with the timeline used for the definition of chronic postsurgical pain, which typically requires pain persistence at this stage to be considered chronic^22^.

Anonymized patient records were obtained from two previously validated^23^ linked databases: primary Clinical Practice Research Datalink (CPRD) Aurum^24^ and linked secondary care Hospital Episode Statistics (HES)^25^ data as previously described^8^. The HES database contains details of all admissions to NHS hospitals in England. Surgery records are coded within the database using the Office of Population Censuses and Surveys (OPCS 4) Classification of Surgical Operations and Procedures (https://www.datadictionary.nhs.uk/supporting_information/opcs_classification_of_interventions_and_procedures.html). Other medical diagnoses are coded within HES using International Classification of Diseases 10th revision (ICD-10) codes (https://icd.who.int/browse10/2019/en). CPRD Aurum contains routinely collected data from general practitioner (GP) practices. In the UK, 98% of the population is registered with a GP, the gatekeeper of care in the NHS. Data provided by CPRD Aurum captures diagnoses, prescriptions, tests and referrals for 7 million active patients. Links to the Office for National Statistics data helped obtain the date of death.

Patients ≥18 years of age who underwent colectomy between 1 January 2010 and 31 December 2019 were identified from HES using OPCS codes for colectomy procedures (*Table S1*). The validity and reliability of codes to identify colectomy have been confirmed previously^23^. Patients were excluded if they did not have at least 12 months CPRD Aurum data before their date of surgery, to ensure complete preoperative opioid exposure data.

Patients issued opioid prescriptions were identified based on the presence of a prescription for an opioid within 90 days of hospital discharge after surgery. Opioid prescription records were identified using opioid product codes^8^ (*Table S2*) and extracted from the CPRD Aurum database then prepared for analysis using an adapted version of the DrugPrep algorithm which allows for identifying prescription errors, duplicate records and dealing with missing data (*Table S3*)^26^. Excluded opioids included higher-strength buprenorphine sublingual tablets (2, 4 and 8 mg) and methadone because they are primarily prescribed for opioid dependence in the UK. Injectable formulations were also excluded as these are typically administered by healthcare professionals rather than self-administered^8^.

The primary outcome was the proportion of patients having an initial opioid prescription issued from general practice within 90 days of hospital discharge following colectomy. For this outcome, the method described previously^8^ was employed to stratify the population based on opioid exposure before colectomy into three groups (opioid naive, currently exposed and previously exposed). This stratification enabled the impact of previous opioid exposure on postoperative opioid use to be described, and whether there were any differences in outcome between groups to be determined. Patients were categorized as opioid naive if they did not receive an opioid prescription in the year leading up to their surgical admission. Those who received an opioid prescription within the 6 months before their admission date were considered ‘currently exposed’, while those who received an opioid prescription within 7 to 12 months prior to admission were classified as ‘previously exposed’, forming two separate preoperative groups with no overlap^8,27^.

Several secondary outcomes were investigated. First, the potency of opioids in the initial prescription: opioids were classified as weak (codeine, dihydrocodeine, meptazinol, pentazocine, tramadol) or strong (morphine, oxycodone, fentanyl, buprenorphine, hydromorphone, pethidine, naloxone/oxycodone, cyclizine/dipipanone, hydromorphone, tapentadol) or a combination of both^28^. Tramadol can be classed as a strong^29^ or weak opioid^28^, and to allow comparison with other UK studies^30,31^ it was classified as a weak opioid.

Second, the opioid prescribed was determined as defined as the drug class (buprenorphine, codeine, dihydrocodeine, fentanyl, morphine, oxycodone, tramadol and other opioids). ‘Other opioids’ represent opioids that were not commonly prescribed and combined in one category during drug preparation. Third, opioid formulations were categorized into immediate-release only, modified-release only, both immediate- and modified-release, transdermal only and others. Fourth, the amount of opioid in each prescription was described as the OMEQ dose in mg/day. The OMEQ dose was used to convert the doses of different opioids into a standard unit based on their analgesic potency to provide more easily comparable data across a range of opioid medicines. It warrants consideration as a standard prescribing measure^32^ and is calculated by multiplying the daily dose of opioids in each prescription by the equivalent analgesic ratio as specified by the US Centers for Disease Control and Prevention^33^ (*Table S4*). For those on a combination of opioids, the OMEQ dose was calculated for each drug and combined to provide an overall OMEQ dose in mg/day for each patient, and was categorized into dose ranks: ≤ 24, 25–49, 50–99, 100–249 and ≥250 mg/day.

Patient characteristics, including sex and age at the time of admission, ethnicity and co-morbidities were ascertained. Ethnic groups were classified as white, Asian, black and others. The Charlson Co-morbidity Index was used to group patients, based on the number of co-morbidities, into categories of 0, 1 and ≥2. Admission type was categorized as emergency or elective according to the recorded admission classification for the surgical procedure. The patients’ level of deprivation was determined using 2015 Index of Multiple Deprivation (IMD) scores^34^, categorized into quintiles ranging from 1 (most deprived) to 5 (least deprived). The surgical technique was categorized as either open or minimally invasive. Minimally invasive surgery included laparoscopic or robotic techniques and was identified using procedural codes (Y50.8, Y57.1, Y75.2, Y75.3). Patients were recorded as having benign disease if the ICD-10 discharge codes for their hospital admission indicated diverticular disease or inflammatory bowel disease. A diagnosis of cancer was confirmed if patients had a recorded diagnosis of colorectal cancer.

Data management and analyses were performed using Stata® version 17 (StataCorp, College Station, TX, USA). Characteristics of the colectomy population over time are presented as proportions for each year. For the primary outcome of the annual proportion of people receiving an opioid prescription, the analysis was stratified based on opioid exposure prior to colectomy. This was calculated as a % for each year:


$$\frac{\begin{array}{c} \mathrm{Number}\;\mathrm{of}\;\mathrm{individuals}\;\mathrm{receiving}\;\mathrm{at}\;\mathrm{least}\;\mathrm{one}\;\mathrm{opioid}\;\mathrm{prescription}\\ \mathrm{within}\;90\;\mathrm{days}\;\mathrm{of}\;\mathrm{discharge}\;\mathrm{in}\;\mathrm{the}\;\mathrm{year}\times 100 \end{array}}{\mathrm{Number}\;\mathrm{of}\;\mathrm{individuals}\;\mathrm{who}\;\mathrm{had}\;\mathrm{colectomy}\;\mathrm{that}\;\mathrm{year}\;}$$

Trend analysis over the years was performed using the Cochran Armitage test. Absolute and % changes between 2010 and 2019 were calculated and tested using unadjusted logistic regression.

For secondary outcomes, characteristics of patients having opioid prescriptions within 90 days of discharge are presented as proportions for each year. Descriptive statistics were used to describe the outcome measures as frequencies, proportions (%).

The yearly proportion of prescriptions containing an opioid formulation was calculated:


$$\frac{\mathrm{Number}\;\mathrm{of}\;\mathrm{prescriptions}\;\mathrm{in}\;\mathrm{each}\;\mathrm{opioid}\;\mathrm{drug}\;\mathrm{formulation}\;\mathrm{category}\;}{\begin{array}{c} \mathrm{Total}\;\mathrm{number}\;\mathrm{of}\;\mathrm{prescriptions}\;\mathrm{for}\;\mathrm{opioids}\;\mathrm{within}\\ \mathrm{first}\;90\;\mathrm{days}\;\mathrm{of}\;\mathrm{discharge}\;\mathrm{in}\;\mathrm{that}\;\mathrm{year} \end{array}}$$

The proportion of people who were dispensed an OMEQ dose in each category was calculated and the proportion of people prescribed an opioid in each category was calculated by dividing the number of people based on their daily OMEQ dose by the total number of people with repeated opioid prescriptions following surgery for that year. Utilization measures were stratified based on opioid exposure before surgery into naive, currently and previously exposed.

## Results

**Fig. 1:**
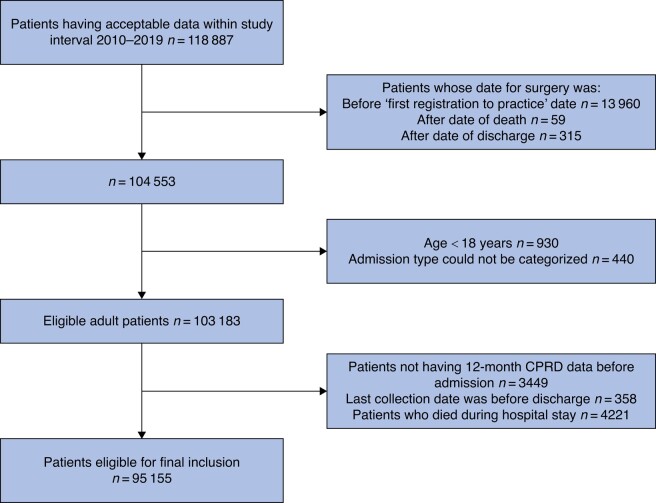
Study flow diagram CPRD, Clinical Practice Research Datalink.

In total, 95 155 individuals had a colectomy during the study interval and met the inclusion criteria (*Fig. 1*). Over the study interval, the surgical approach shifted toward open colectomy being less frequent than minimally invasive procedures. There was an increase in the number of opioid-naive patients from 79.1% in 2010 to 86.9% in 2019 (% change +9.9%, *P* <0.005), whereas there was a fall in the currently opioid-exposed group (% change −40.5%, *P* <0.001) (*Table S5*).

Of 15 503 (16.3%) individuals who received opioid prescriptions from primary care after hospital discharge following colectomy, the ratio of opioid-naive to currently exposed individuals and elective to emergency admission remained relatively stable over the study interval. However, the proportions of individuals having two or more co-morbidities, benign disease and minimally invasive procedures increased over the study interval (*Table 1*).

**Table 1:** Baseline characteristics of patients having opioid prescriptions within 90 days following colectomy discharge between the years 2010 and 2019

Variable	Years									
	2010	2011	2012	2013	2014	2015	2016	2017	2018	2019
**Whole colectomy**	8001	8470	8850	8963	9038	9614	10 009	10 286	10 794	11 130
**Issued initial opioid prescription**	1607 (100%)	1689 (100%)	1663 (100%)	1653 (100%)	1537 (100%)	1566 (100%)	1466 (100%)	1489 (100%)	1435 (100%)	1398 (100%)
**Age (years), mean (s.d.)**	65.2 (14.3)	64.9 (14.3)	64.1 (15.3)	63.7 (15.2)	64.5 (15.0)	63.7 (15.2)	63.5 (15.4)	63.4 (15.4)	63.2 (14.8)	63.4 (15.4)
**Sex**										
Female	850 (52.9)	843 (49.9)	886 (53.3)	892 (54.0)	821 (53.4)	831 (53.1)	808 (55.1)	811 (54.5)	779 (54.3)	773 (55.3)
Male	757 (47.1)	846 (50.1)	777 (46.7)	761 (46.0)	716 (46.6)	735 (46.9)	658 (44.9)	678 (45.5)	656 (45.7)	625 (44.7)
**Ethnicity**										
White	1530 (95.2)	1600 (94.7)	1563 (93.9)	1560 (94.3)	1437 (93.5)	1452 (92.7)	1355 (92.4)	1370 (92.0)	1328 (92.5)	1258 (90.0)
Black	32 (1.9)	22 (1.3)	21 (1.3)	25 (1.5)	20 (1.3)	33 (2.1)	26 (1.8)	30 (2.01)	32 (2.2)	34 (2.4)
Asian	36 (2.2)	41 (2.4)	47 (2.8)	40 (2.4)	40 (2.6)	37 (2.4)	40 (2.7)	38 (2.6)	32 (2.2)	49 (3.5)
Others	9 (0.56)	26 (1.5)	32 (1.9)	28 (1.7)	40 (2.6)	44 (2.8)	45 (3.1)	51 (3.4)	43 (3.0)	57 (4.1)
**Index of Multiple Deprivation (1 most deprived, 5 least deprived)**										
1	315 (19.6)	338 (20.0)	316 (19.0)	323 (19.5)	298 (19.4)	324 (20.7)	276 (18.8)	305 (20.5)	305 (21.3)	265 (18.9)
2	335 (20.9)	343 (20.3)	334 (20.1)	349 (21.1)	311 (20.2)	305 (19.5)	295 (20.1)	293 (19.7)	258 (17.9)	270 (19.3)
3	348 (21.7)	325 (19.2)	326 (19.6)	316 (19.1)	318 (20.7)	315 (20.1)	330 (22.5)	289 (19.4)	298 (20.8)	285 (20.4)
4	306 (19.0)	347 (20.5)	324 (19.5)	351 (21.2)	325 (21.2)	291 (18.6)	299 (20.4)	289 (19.4)	282 (19.7)	275 (19.6)
5	301 (18.7)	332 (19.7)	363 (21.8)	314 (19.0)	283 (18.4)	330 (21.1)	263 (17.9)	311 (20.9)	291 (20.3)	303 (21.7)
Missing	2 (0.12)	4 (0.24)	–	–	–	–	3 (0.20)	2 (0.13)	1 (0.07)	–
**Charlson Co-morbidity Index**										
0	261 (16.3)	252 (14.9)	279 (16.8)	282 (17.1)	248 (16.1)	262 (16.7)	220 (15.0)	204 (13.7)	206 (14.3)	198 (14.2)
1	119 (7.4)	120 (7.1)	110 (6.6)	104 (6.3)	99 (6.4)	103 (6.6)	88 (6.0)	114 (7.7)	87 (6.1)	82 (5.9)
≥2	1227 (76.4)	1317 (77.9)	1274 (76.6)	1267 (76.7)	1190 (77.4)	1201 (76.7)	1158 (78.9)	1171 (78.6)	1142 (79.6)	1118 (79.9)
**Preoperative opioid exposure**										
Opioid naive	721 (44.9)	804 (47.6)	774 (46.5)	752 (45.5)	707 (46.0)	695 (44.4)	642 (43.8)	632 (42.5)	607 (42.3)	647 (46.3)
Currently exposed	808 (50.3)	795 (47.1)	800 (48.1)	823 (49.8)	755 (49.1)	794 (50.7)	753 (51.4)	789 (52.3)	753 (52.5)	677 (48.4)
Previously exposed	78 (4.9)	90 (5.3)	89 (5.4)	78 (4.7)	75 (4.9)	77 (4.9)	71 (4.8)	68 (4.6)	75 (5.2)	74 (5.3)
**Surgical approach**										
Open	1211 (75.4)	1259 (74.5)	1196 (71.9)	1140 (68.9)	1032 (67.1)	1026 (65.5)	932 (63.6)	900 (60.4)	831 (57.9)	781 (55.9)
Minimally invasive	396 (24.6)	430 (25.5)	467 (28.1)	513 (31.0)	505 (32.9)	540 (34.5)	534 (36.4)	589 (39.6)	604 (42.1)	617 (44.1)
**Cancer diagnosis**										
No	680 (42.3)	656 (38.8)	756 (45.5)	734 (44.4)	685 (44.6)	699 (44.6)	657 (44.8)	681 (45.7)	653 (45.5)	662 (47.4)
Yes	927 (57.7)	1033 (61.2)	907 (54.5)	919 (55.6)	852 (55.4)	867 (55.4)	809 (55.2)	808 (54.3)	782 (54.5)	736 (52.6)
**Admission type**										
Emergency	453 (28.2)	497 (29.4)	531 (31.9)	526 (31.8)	486 (31.6)	503 (32.1)	464 (31.7)	452 (30.4)	438 (30.5)	468 (33.5)
Elective	1154 (71.8)	1192 (70.6)	1132 (68.1)	1127 (68.2)	1051 (68.4)	1063 (67.9)	1002 (68.4)	1037 (69.6)	997 (69.5)	930 (66.5)

Values are *n* (%) unless otherwise stated.

**Fig. 2:**
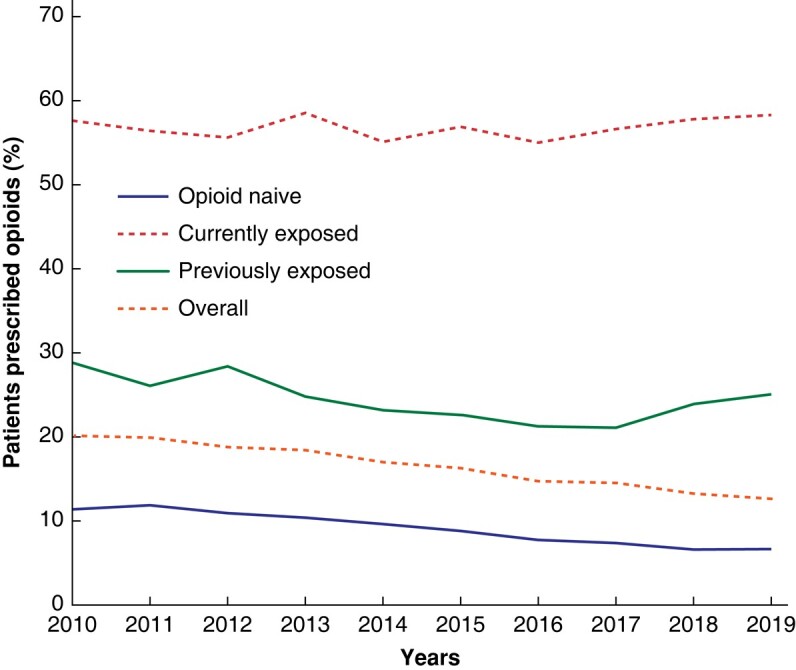
Temporal trend of percentage of patients who received opioid prescriptions after discharge

Overall, the percentage of patients issued an opioid prescription within 90 days of hospital discharge decreased by 37.3% between 2010 and 2019 (*P* <0.001). The opioid-naive group mainly drove this downward trend, with the proportion of people prescribed any opioid decreasing from 11.4% in 2010 to 6.7% in 2019 (% change −41.3%, *P* <0.001), whereas, for the currently exposed group, the percentage of individuals prescribed opioids remained stable, from 57.5% in 2010 to 58.3% in 2019 (% change +1.3%, *P* = 0.697) (*Fig. 2*).

**Fig. 3:**
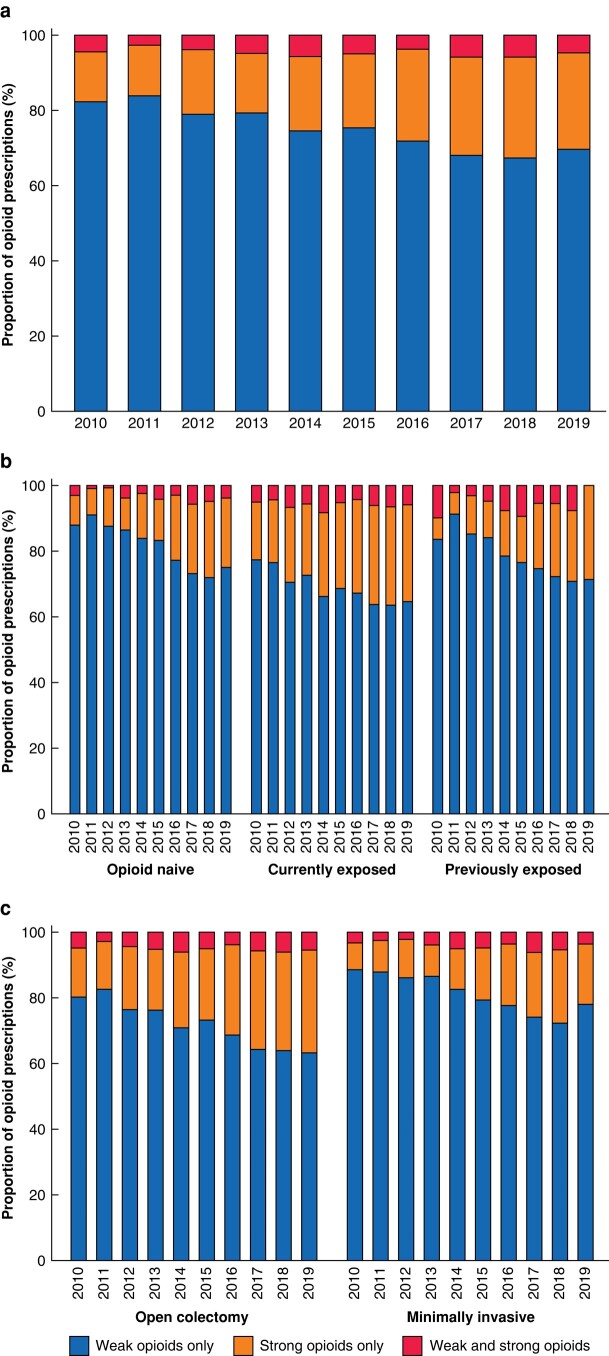
Yearly trend in the potency of opioid prescribed in initial prescription received after discharge **a** overall cohort, **b** cohort stratified by opioid exposure before colectomy and **c** cohort stratified by surgical approach.

For trends in opioid prescriptions by opioid potency, weak opioids were the most commonly prescribed category during the study interval (75.5%), followed by strong opioids (19.9%), with the remainder (4.6%) prescribed a combination of weak and strong opioids. Notably, there was a downward trend in weak opioid prescribing prevalence over the years, with a decline from 82.3% in 2010 to 69.7% in 2019 (% change −15.3%, *P* <0.001). This decline remained statistically significant for all three strata of previous opioid exposure (*P* <0.001), and for both open and minimally invasive surgeries (*P* <0.001) (*Fig. 3* and *Table S6*).

There was an upward trend in the prescribing prevalence of strong opioids, with a 94.0% increase (13.2% in 2010 to 25.6% in 2019, *P* <0.001). In addition, strong opioid prescribing was more common for currently opioid-exposed (64.3%) than for the opioid-naive (31.9%) and previously exposed groups (3.7%). However, the temporal changes were steeper for the opioid-naive group, with a 133% increase from 2010 to 2019. Although strong opioid prescribing increased at a similar rate for both open and minimally invasive colectomy (15.0% in 2010 to 31.0% in 2019 (*P* <0.001) and 8.1% in 2010 to 18.3% in 2019 (*P* <0.001) respectively), strong opioid prescribing remained lower in the minimally invasive colectomy group than in those having open colectomy (*Fig. 3* and *Table S6*).

**Fig. 4:**
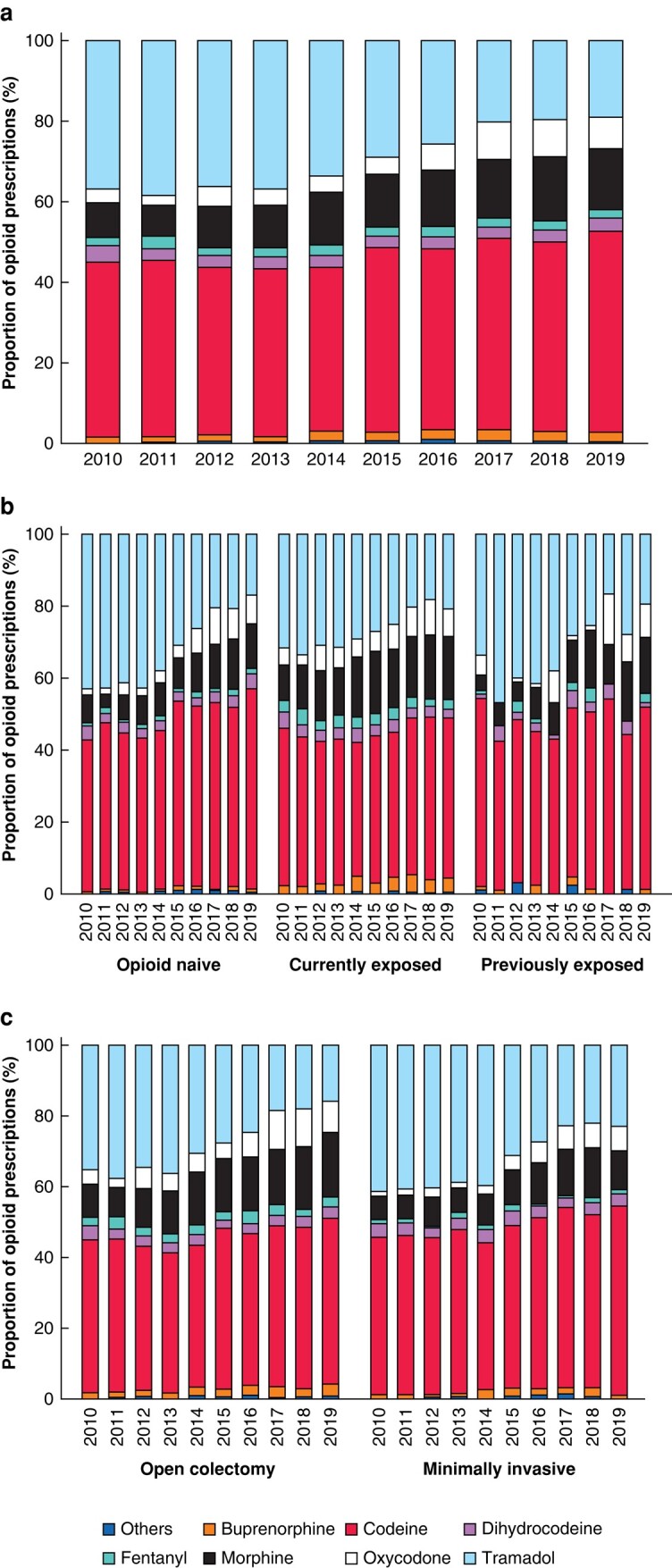
Yearly trend in the type of opioid prescribed in initial prescription received after discharge **a** overall cohort, **b** cohort stratified by opioid exposure before colectomy and **c** cohort stratified by surgical approach.

Codeine was the most commonly prescribed opioid during the study interval (44.5%), followed by tramadol (29.9%) and morphine (12.2%). Notably, prescribing of specific opioid medicines changed over time. For the overall population, the prescribing of codeine decreased from 43.5% in 2010 to 40% in 2014 and rose to 50.0% in 2019 (*P* <0.001). When people were stratified either by surgical approach or by opioid exposure before surgery, codeine remained the most commonly prescribed opioid. However, the significantly increased prescribing prevalence was mainly for the opioid-naive group and minimally invasive surgery (*Fig. 4* and *Table S7*).

The proportion of prescriptions for oxycodone and morphine continued to increase. Oxycodone prescribing nearly trebled between 2010 and 2019 for the opioid-naive group and minimally invasive surgery (% change: +395% and +471% respectively). On the contrary, buprenorphine prescribing increased from 2.2% to 4.1% for the currently opioid-exposed group and from 4.1% to 8.6% for open colectomy. Among all the prescribed opioids, tramadol prescribing decreased significantly, with a steep decline starting in 2014. This decline was evident for all stratified groups.

Overall, immediate-release formulations were the most prescribed formulation over the study interval (83.9%), followed by modified-release only (5.8%) and transdermal only (3.1%). Although immediate-release formulations were more prominent, their prevalence decreased from 86.0% in 2010 to 82.0% in 2019 (% change −4.7%, *P* <0.001). Transdermal and modified-release formulations were prescribed more for the currently exposed group (76.8% and 69.0% respectively), compared with 20.0% and 27.6% respectively in the opioid-naive group.

**Fig. 5:**
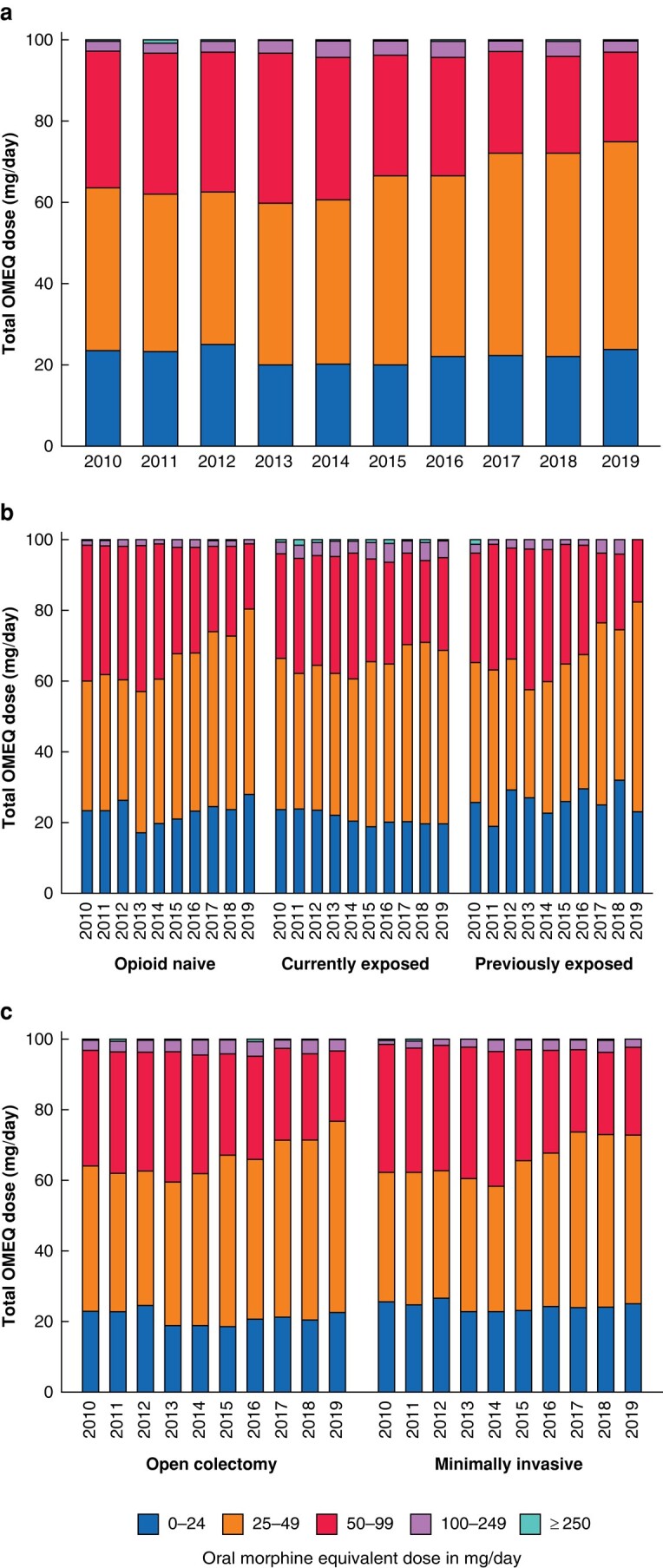
Yearly trend in total oral morphine equivalent (OMEQ) doses prescribed in initial prescription received after discharge **a** overall cohort, **b** cohort stratified by opioid exposure before colectomy and **c** cohort stratified by surgical approach.

Of the five dose ranks of total OMEQ dose, most people were in the lower dose ranks (43.6% in the 25–49 mg/day group and 30.8% in the 50–99 mg/day group). There was an increasing trend in the percentage of people prescribed opioids in the 25–49 mg/day group from 40.1% to 51.3% (*P* <0.001), with the increase being more predominant for the opioid-naive and prior exposed groups. On the other hand, a downward trend, which started in 2013, was seen for doses of 50–99 mg/day (% change −34.5%) (*Fig. 5*).

## Discussion

There was a notable decrease in patients receiving opioid prescriptions in primary care following discharge after colectomy, particularly among opioid-naive patients over time. These results are consistent with those of studies from the USA that also showed a decline in opioid prescribing after major abdominal and orthopaedic surgeries^18,19^, despite the differences between postsurgical prescribing practices in the USA and the UK. It is essential to note that due to a dearth of studies on postoperative opioid prescribing practices in the UK, direct comparisons with current study findings are limited.

Codeine and tramadol were the most frequently prescribed opioids after colectomy, consistent with findings that codeine and tramadol accounted for approximately 58% and 45% of postoperative prescriptions in Canada and Sweden respectively, compared with only 7% in the USA^4^. A similar trend was observed in a cross-sectional study that examined the type of opioid initiated for new users in different countries, where a higher proportion of patients was started on codeine and tramadol in the UK, while oxycodone was the most commonly prescribed opioid in the USA^35^.

The present study also found a decrease in prescribing of tramadol after 2014, reaching a similar rate to morphine prescribing by the end of the study interval. One possible explanation for this decline is the classification of tramadol as a Schedule 3 controlled substance in the UK in 2014^36^, prompted by safety concerns and potential risks of misuse. This decrease in tramadol prescribing is consistent with a UK study that assessed the impact of reclassification on the use of tramadol for chronic pain^37^. Another observed change over time was the increase in prescribing of oxycodone and morphine, consistent with trends reported in contemporary prescribing literature from the UK for other indications^35,38^.

While the data included in this study were obtained before the release of guidelines that advise against using transdermal and long-acting formulations for managing acute pain^2^, the findings are reassuring since immediate-release formulations accounted for most prescribed opioids. However, the unexpected modest decrease in the prescribing of immediate-release formulations in 2019 is noteworthy. It is also worth emphasizing that prescribing transdermal formulations was more common among patients with previous opioid exposure, which could be attributed to the continuation of similar formulations after hospital discharge. Nevertheless, increased education and awareness are necessary to discourage the use of long-acting formulations in favour of immediate-release opioids due to their higher risk of misuse, addiction and difficulty in dose adjustment.

Most patients were prescribed low opioid doses (OMEQ dose in the 25–49 and 59–99 mg/day categories), with an upward trend for patients prescribed an OMEQ dose in the 25–49 mg/day category over the years. International guidelines vary in the OMEQ doses that require caution. Canadian guidelines recommend that the prescribed OMEQ dose should be limited to <50 mg/day^39^, while US guidelines advise prescribers to avoid increasing the dose to ≥90 mg/day^33^. The UK Faculty of Pain Medicine advises that the potential harms outweigh the benefits when an OMEQ dose of 120 mg/day is exceeded^40^. It is challenging to identify any specific intervention or policy that contributed to the trends in prescribing observed in this study. Possible interventions could be the improvement of perioperative pain management approaches and surgical techniques, particularly since there was an increase in the adoption of minimally invasive surgeries in this cohort. Other contributing factors include the promotion of non-opioid analgesia or opioid-sparing strategies, and the availability of patient-provider education and discharge counselling services^8^.

Several clinical implications arise from the findings of this study. First, although the UK guidelines on perioperative opioid prescribing^22,41^ do not provide metrics on the proportion of patients on opioids to indicate best practice, the study findings may indicate a potential decrease in the reliance on opioids for managing acute pain following colectomy in England and could represent a trend towards improved opioid stewardship. Second, the consensus guidelines on preventing opioid-related harm suggest that postdischarge repeat prescriptions for opioids should be avoided, given the potential risks involved^2,41^. This recommendation becomes particularly relevant as the current analysis showed that some patients still need opioids within 90 days after discharge, which might raise concerns about the possibility of developing chronic postsurgical pain. As opioids are not recommended for managing chronic postsurgical pain^2^, requests for additional opioids should prompt a comprehensive patient review by the GP or pain specialists for opioid weaning or assessment of chronic postsurgical pain^42,43^. Since the data used were collected before the release of these guidelines, future studies should evaluate the impact of guideline implementation on reducing opioid prescribing and opioid-related adverse events for surgical patients.

Third, the study showed that codeine and tramadol were the most commonly prescribed opioids. Despite being classified as weak opioids, being prodrugs both can have different side effect profiles based on individual genetic polymorphisms^44^. The classification of opioids based on potency has been debated^7^ as this alone does not protect patients from potential harm, including dependence and death^45^. Notably, while codeine-related deaths in the UK increased by 17-fold from 9 in 1994 to 156 in 2017^46^, the drug is still available over-the-counter. In contrast, the Australian Federal Government reclassified codeine as a prescription-only medicine in 2018, resulting in a subsequent reduction in harm associated with its use^47,48^. The results of the present study can indicate possible opportunities to re-evaluate analgesic selection practices and educate healthcare professionals about the variable effects and side effects profile of different opioids.

This study had some limitations. While it describes trends and patterns in opioid use, it did not assess the specific factors at the patient, provider or system level that influenced them. Accuracy of recorded data is a common concern when using electronic health records. Nevertheless, the databases used in this study have undergone thorough validation and have implemented various measures to ensure data quality and accuracy. Moreover, the opioid prescription data were prepared using a systematic approach with a prescription preparation algorithm. This algorithm addresses missing data, accounts for overlapped prescriptions and calculates the OMEQ dose, allowing for more easily comparable data across different opioid medications since the OMEQ dose is considered a standard prescribing measure.

Another shortcoming of electronic health record data is the lack of detailed clinical contexts, such as specific medication-use indications, patient preferences and clinical decision-making processes. This limitation also applies to analgesics prescribed during hospital stay, which may impact the choice of opioid and prescribed doses. For example, the use of adjunctive paracetamol or non-steroidal anti-inflammatory drugs immediately after surgery is associated with decreased postoperative opioid requirements and reduced related adverse events^49^. Finally, while using prescription data as a proxy measure of drug consumption is a well established practice in drug utilization research^9^, it is essential to acknowledge its limitations. In the present study, the availability of issued opioid prescriptions was used as a surrogate marker to ascertain opioid consumption. However, as information on actual consumption and adherence was lacking, overall utilization may have been overestimated.

## Supplementary Material

zrad136_Supplementary_Data

## Data Availability

Access to the Clinical Practice Research Datalink and linked data was provided under a licence to the University of Nottingham. Under the terms and agreement of this licence, data cannot be provided for the purposes of sharing.
