# Supplementary material for: Temporal trends and patterns in initial opioid prescriptions after hospital discharge following colectomy in England over 10 years
Source: BJS Open. 2023 Dec 26;7(6):zrad136. doi: 10.1093/bjsopen/zrad136 (PMC10750262; doi:10.1093/bjsopen/zrad136)
Supplement: zrad136_Supplementary_Data [file zrad136_supplementary_data.docx]

**Original Article**

**Temporal trends and patterns in initial opioid prescriptions after hospital discharge following colectomy in England over ten years**

Reham M. Baamer^1,2^, David J. Humes^3,4^, Li Shean Toh^1^, Roger D. Knaggs^1,5^, Dileep N. Lobo^3,4,6,7^

^1^Division of Pharmacy Practice and Policy, School of Pharmacy, University of Nottingham, Nottingham, UK

^2^Department of Pharmacy Practice, Faculty of Pharmacy, King Abdulaziz University, Jeddah, Saudi Arabia

^3^Nottingham Digestive Diseases Centre, Division of Translational Medical Sciences, School of Medicine, University of Nottingham, Queen’s Medical Centre, Nottingham, UK

^4^National Institute for Health Research Nottingham Biomedical Research Centre, Nottingham University Hospitals NHS Trust and University of Nottingham, Queen’s Medical Centre, Nottingham, UK

^5^Pain Centre Versus Arthritis, University of Nottingham, Nottingham, UK

^6^David Greenfield Metabolic Physiology Unit, MRC Versus Arthritis Centre for Musculoskeletal Ageing Research, School of Life Sciences, University of Nottingham, Queen’s Medical Centre, Nottingham, UK

^7^Department of Surgery, Perelman School of Medicine, University of Pennsylvania, Philadelphia, PA, USA

Correspondence to:

Prof. D. N. Lobo

Nottingham Digestive Diseases Centre

E Floor, West Block

Nottingham University Hospitals

Queen’s Medical Centre

Nottingham NG7 2UH

UK

Fax: +44-115-8231160

Email: [dileep.lobo@nottingham.ac.uk](mailto:Dileep.Lobo@nottingham.ac.uk)

**ORCID ID**: 0000-0003-1187-5796

**Supplementary Materials - Index**

| **Supplementary Figures and Tables** |  |
| --- | --- |
| Table S1: HES using OPCS codes for colectomy procedures | *pp. 3-5* |
| Table S2: Opioid products codes | *pp.6-40* |
| Table S3: Decisions made to prepare opioid prescriptions using DrugPrep algorithm | *pp. 41-42* |
| Table S4: **Equianalgesic ratios to calculate Oral Morphine Equivalent dose** | *pg. 43* |
| Table S5: Yearly characteristics of the colectomy cohort | *pp. 44-45* |
| Table S6: Changes in the potency of opioid prescribed in initial prescription received after discharge | *pp. 46-48* |
| Table S7: Changes in the type of opioid prescribed in initial prescription received after discharge | *pp. 49-50* |

**Supplementary Materials**

**Table S1: OPCS and ICD codes used to identify colectomy, inflammatory bowel disease and diverticular disease**

**Colectomy codes**

**H04 Total excision of colon and rectum *(Clean-Contaminated)***

H04.1 Panproctocolectomy and ileostomy

*Includes: Proctocolectomy not elsewhere classified*

H04.2 Panproctocolectomy and anastomosis of ileum to anus and creation of pouch however further qualified

H04.3 Panproctocolectomy and anastomosis of ileum to anus not elsewhere classified

H04.8 Other specified

H04.9 Unspecified

**H05 Total excision of colon *(Clean-Contaminated*)**

H05.1 Total colectomy and anastomosis of ileum to rectum

H05.2 Total colectomy and ileostomy and creation of rectal fistula however further qualified

H05.3 Total colectomy and ileostomy not elsewhere classified

H05.8 Other specified

H05.9 Unspecified

**H06 Extended excision of right hemicolon *(Clean-Contaminated)***

*Includes: Excision of right colon and other segment of ileum or colon and surrounding tissue*

H06.1 Extended right hemicolectomy and end to end anastomosis

H06.2 Extended right hemicolectomy and anastomosis of ileum to colon

H06.3 Extended right hemicolectomy and anastomosis not elsewhere classified

H06.4 Extended right hemicolectomy and ileostomy however further qualified

H06.5 Extended right hemicolectomy and end to side anastomosis

H06.8 Other specified

H06.9 Unspecified

**H07 Other excision of right hemicolon *(Clean-Contaminated*)**

*Includes: Limited excision of caecum and terminal ileum caecum*

H07.1 Right hemicolectomy and end to end anastomosis of ileum to colon

*Includes: Ileocaecal resection*

H07.2 Right hemicolectomy and side to side anastomosis of ileum to transverse colon

H07.3 Right hemicolectomy and anastomosis not elsewhere classified

H07.4 Right hemicolectomy and ileostomy however further qualified

H07.5 Right hemicolectomy and end to side anastomosis

H07.8 Other specified

H07.9 Unspecified

**H08 Excision of transverse colon *(Clean-Contaminated)***

H08.1 Transverse colectomy and end to end anastomosis

H08.2 Transverse colectomy and anastomosis of ileum to colon

H08.3 Transverse colectomy and anastomosis not elsewhere classified

H08.4 Transverse colectomy and ileostomy however further qualified

H08.5 Transverse colectomy and exteriorisation of bowel not elsewhere classified*

H08.6 Transverse colectomy and end to side anastomosis

H08.8 Other specified

H08.9 Unspecified

****Note:*** *Use secondary code for exteriorisation of caecum (H14) or other exteriorisation of colon (H15)*

**H09 Excision of left hemicolon (*Clean-Contaminated)***

H09.1 Left hemicolectomy and end to end anastomosis of colon to rectum

H09.2 Left hemicolectomy and end to end anastomosis of colon to colon

H09.3 Left hemicolectomy and anastomosis not elsewhere classified

H09.4 Left hemicolectomy and ileostomy however further qualified

H09.5 Left hemicolectomy and exteriorisation of bowel not elsewhere classified*

H09.6 Left hemicolectomy and end to side anastomosis

H09.8 Other specified

H09.9 Unspecified

****Note:*** *Use secondary code for exteriorisation of caecum (H14) or other exteriorisation of colon (H15)*

**H10 Excision of sigmoid colon (*Clean-Contaminated)***

H10.1 Sigmoid colectomy and end to end anastomosis of ileum to rectum

H10.2 Sigmoid colectomy and anastomosis of colon to rectum

H10.3 Sigmoid colectomy and anastomosis not elsewhere classified

H10.4 Sigmoid colectomy and ileostomy however further qualified

H10.5 Sigmoid colectomy and exteriorisation of bowel not elsewhere classified*

H10.6 Sigmoid colectomy and end to side anastomosis

H10.8 Other specified

H10.9 Unspecified

****Note:*** *Use secondary code for exteriorisation of caecum (H14) or other exteriorisation of colon (H15)*

**H11 Other excision of colon *(Clean-Contaminated)***

*Includes: Excision of colon where segment removed is not stated*

H11.1 Colectomy and end to end anastomosis of colon to colon not elsewhere classified SSI H11.2 Colectomy and side to side anastomosis of ileum to colon not elsewhere classified

H11.3 Colectomy and anastomosis not elsewhere classified

H11.4 Colectomy and ileostomy not elsewhere classified

H11.5 Colectomy and exteriorisation of bowel not elsewhere classified*

H11.6 Colectomy and end to side anastomosis NEC

H11.8 Other specified

**Please see minimum wound class against each procedure**

H11.9 Unspecified

*Includes: Colectomy or hemicolectomy not elsewhere classified*

****Note****: Use secondary code for exteriorisation of caecum (H14) or other exteriorisation of colon (H15)*

**H29 Subtotal excision of colon and rectum *(Clean contaminated)***

H29.1 Subtotal excision of colon and rectum and creation of colonic pouch and anastomosis of colon to anus

H29.2 Subtotal excision of colon and rectum and creation of colonic pouch NEC

H29.3 Subtotal excision of colon and creation of colonic pouch and anastomosis of colon to rectum.

H29.4 Subtotal excision of colon and creation of colonic pouch NEC

H29.8 Other specified subtotal excision of colon

H29.9 Unspecified subtotal excision of colon

**H33 Excision of rectum *(Clean contaminated)***

*Includes: Excision of whole or part of rectum with or without part of sigmoid colon*

H33.1 Abdominoperineal excision of rectum and end colostomy

H33.2 Proctectomy and anastomosis of colon to anus

H33.3 Anterior resection of rectum and anastomosis of colon to rectum using staples

*Includes: Rectosigmoidectomy and anastomosis of colon to rectum*

H33.4 Anterior resection of rectum and anastomosis not elsewhere classified

H33.5 Rectosigmoidectomy and closure of rectal stump and exteriorisation of bowel*

H33.6 Anterior resection of rectum and exteriorisation of bowel*

H33.7 Perineal resection of rectum HFQ

H33.8 Other specified

H33.9 Unspecified

*Includes: Rectosigmoidectomy not elsewhere classified*

****Note:*** *Use secondary code for creation of artificial opening into ileum (G74); exteriorisation of caecum (H14) or other exteriorisation of colon (H15*

**Inflammatory bowel disease codes**

K50, K500, K501, K508, K509, K51, K510, K512, K513, K514, K515, K518, K519, K520, K521, K522, K523, K528, K529

**Diverticular disease codes**

K57,K570, K571, K572, K573, K574, K575, K578, K579

**Table S2: Opioid products code**

| ProdCodeId | DMDCode | drugsubstancename | substancestrength | formulation |
| --- | --- | --- | --- | --- |
| 1.23539E+16 | 3.45353E+16 | Tramadol hydrochloride | 75mg | Modified-release tablet |
| 1.23538E+16 | 3.45363E+16 | Tramadol hydrochloride | 200 mg | Modified-release tablet |
| 1.23537E+16 | 3.45349E+16 | Tramadol hydrochloride | 150 mg | Modified-release tablet |
| 1.23536E+16 | 3.45358E+16 | Tramadol hydrochloride | 100 mg | Modified-release tablet |
| 1.85044E+15 | 4.61411E+14 | Tramadol hydrochloride | 400 mg | Modified-release tablet |
| 1.85034E+15 | 9.29211E+14 | Tramadol hydrochloride | 300 mg | Modified-release tablet |
| 1.85024E+15 | 1.42111E+14 | Tramadol hydrochloride | 200 mg | Modified-release tablet |
| 1.85014E+15 | 1.39611E+14 | Tramadol hydrochloride | 150 mg | Modified-release tablet |
| 4.25924E+15 | 1.19853E+16 | Tramadol hydrochloride | 50 mg | Modified-release tablet |
| 1.56474E+15 | 3.15611E+14 | Tramadol hydrochloride | 200 mg | Modified-release tablet |
| 1.56464E+15 | 3.62111E+14 | Tramadol hydrochloride | 150 mg | Modified-release tablet |
| 1.56454E+15 | 3.06311E+14 | Tramadol hydrochloride | 100 mg | Modified-release tablet |
| 1.56484E+15 | 3.14411E+14 | Tramadol hydrochloride | 50 mg | Soluble tablet |
| 1.56434E+15 | 2.03811E+14 | Tramadol hydrochloride | 50 mg | Capsule |
| 1.56154E+15 | 3.77801E+15 | Morphine sulfate | 60 mg | Modified-release capsule |
| 1.56144E+15 | 3.65161E+15 | Morphine sulfate | 30 mg | Modified-release capsule |
| 1.56134E+15 | 4.03551E+15 | Morphine sulfate | 200 mg | Modified-release capsule |
| 1.56114E+15 | 3.65211E+15 | Morphine sulfate | 10 mg | Modified-release capsule |
| 1.56124E+15 | 3.88161E+15 | Morphine sulfate | 100 mg | Modified-release capsule |
| 1.12435E+16 | 3.21335E+16 | Oxycodone hydrochloride | 80 mg | Modified-release tablet |
| 1.12431E+16 | 3.21385E+16 | Oxycodone hydrochloride | 5 mg | Modified-release tablet |
| 1.12434E+16 | 3.21367E+16 | Oxycodone hydrochloride | 40 mg | Modified-release tablet |
| 1.12433E+16 | 3.21402E+16 | Oxycodone hydrochloride | 20 mg | Modified-release tablet |
| 1.12432E+16 | 3.21353E+16 | Oxycodone hydrochloride | 10 mg | Modified-release tablet |
| 4.41754E+15 | 1.28716E+16 | Tramadol hydrochloride | 200 mg | Modified-release tablet |
| 4.41764E+15 | 1.28712E+16 | Tramadol hydrochloride | 150 mg | Modified-release tablet |
| 4.41774E+15 | 1.28698E+16 | Tramadol hydrochloride | 100 mg | Modified-release tablet |
| 2.74614E+15 | 7.5011E+13 | Codeine phosphate/ Paracetamol | 30 mg + 500 mg | Tablet |
| 2.74594E+15 | 3.14111E+14 | Codeine phosphate/ Paracetamol | 30 mg + 500 mg | Capsule |
| 1.54954E+15 | 2.1011E+13 | Tramadol hydrochloride | 50 mg | Modified-release capsule |
| 1.54944E+15 | 9.411E+12 | Tramadol hydrochloride | 200 mg | Modified-release capsule |
| 1.54934E+15 | 7.11511E+14 | Tramadol hydrochloride | 150 mg | Modified-release capsule |
| 1.54924E+15 | 3.31011E+14 | Tramadol hydrochloride | 100 mg | Modified-release capsule |
| 2.98044E+15 | 5.19701E+15 | Tramadol hydrochloride | 50 mg | Orodispersible tablet |
| 1.54804E+15 | 4.00911E+14 | Tramadol hydrochloride | 50 mg | Capsule |
| 3.34444E+15 | 9.53321E+15 | Tramadol hydrochloride | 400 mg | Modified-release tablet |
| 3.34434E+15 | 9.53291E+15 | Tramadol hydrochloride | 300 mg | Modified-release tablet |
| 3.34424E+15 | 9.53261E+15 | Tramadol hydrochloride | 200 mg | Modified-release tablet |
| 3.34414E+15 | 9.53221E+15 | Tramadol hydrochloride | 150 mg | Modified-release tablet |
| 1.0598E+16 | 3.00022E+16 | Fentanyl | 50 microgram/1 hour | Transdermal patch |
| 1.05981E+16 | 3.0002E+16 | Fentanyl | 25 microgram/1 hour | Transdermal patch |
| 1.05982E+16 | 3.00017E+16 | Fentanyl | 12 microgram/1 hour | Transdermal patch |
| 1.05978E+16 | 3.0003E+16 | Fentanyl | 100 microgram/1 hour | Transdermal patch |
| 5.00724E+15 | 1.53636E+16 | Fentanyl | 75 microgram/1 hour | Transdermal patch |
| 5.00754E+15 | 1.53634E+16 | Fentanyl | 50 microgram/1 hour | Transdermal patch |
| 5.00744E+15 | 1.53632E+16 | Fentanyl | 25 microgram/1 hour | Transdermal patch |
| 1.15076E+16 | 3.25202E+16 | Fentanyl | 12 microgram/1 hour | Transdermal patch |
| 5.00734E+15 | 1.53638E+16 | Fentanyl | 100 microgram/1 hour | Transdermal patch |
| 1.47964E+15 | 3.60311E+14 | Codeine phosphate/ Paracetamol | 30 mg + 500 mg | Capsule |
| 1.24286E+16 | 3.49121E+16 | Buprenorphine | 70 microgram/1 hour | Transdermal patch |
| 1.24285E+16 | 3.49119E+16 | Buprenorphine | 52.5 microgram/1 hour | Transdermal patch |
| 1.24284E+16 | 3.49117E+16 | Buprenorphine | 35 microgram/1 hour | Transdermal patch |
| 2.73804E+15 | 3.44931E+15 | Buprenorphine | 70 microgram/1 hour | Transdermal patch |
| 2.73794E+15 | 3.44881E+15 | Buprenorphine | 52.5 microgram/1 hour | Transdermal patch |
| 2.73784E+15 | 3.44661E+15 | Buprenorphine | 35 microgram/1 hour | Transdermal patch |
| 4.45954E+15 | 1.27908E+16 | Tramadol hydrochloride | 200 mg | Modified-release tablet |
| 4.45944E+15 | 1.27905E+16 | Tramadol hydrochloride | 150 mg | Modified-release tablet |
| 4.45934E+15 | 1.27903E+16 | Tramadol hydrochloride | 100 mg | Modified-release tablet |
| 4.52304E+15 | 1.15921E+16 | Tramadol hydrochloride | 50 mg | Modified-release capsule |
| 4.52334E+15 | 1.15927E+16 | Tramadol hydrochloride | 200 mg | Modified-release capsule |
| 4.52324E+15 | 1.15925E+16 | Tramadol hydrochloride | 150 mg | Modified-release capsule |
| 4.52314E+15 | 1.15923E+16 | Tramadol hydrochloride | 100 mg | Modified-release capsule |
| 1.70204E+15 | 3.24291E+15 | Tramadol hydrochloride | 50 mg | Effervescent powder |
| 1.70194E+15 | 3.25011E+15 | Tramadol hydrochloride | 100 mg | Effervescent powder |
| 1.45454E+15 | 4.51511E+14 | Tramadol hydrochloride | 50 mg | Capsule |
| 2.07814E+15 | 3.59212E+16 | Tramadol hydrochloride | 75 mg | Modified-release tablet |
| 1.46574E+15 | 322633008 | Tramadol hydrochloride | 50 mg | Soluble tablet |
| 2.98034E+15 | 5.21281E+15 | Tramadol hydrochloride | 50 mg | Orodispersible tablet |
| 4.25914E+15 | 1.20374E+16 | Tramadol hydrochloride | 50 mg | Modified-release tablet |
| 1.46294E+15 | 3.59401E+16 | Tramadol hydrochloride | 50 mg | Modified-release capsule |
| 1.70224E+15 | 322645004 | Tramadol hydrochloride | 50 mg | Effervescent powder |
| 1.45424E+15 | 322623000 | Tramadol hydrochloride | 50 mg | Capsule |
| 1.85004E+15 | 3.5921E+16 | Tramadol hydrochloride | 400 mg | Modified-release tablet |
| 1.84994E+15 | 3.59209E+16 | Tramadol hydrochloride | 300 mg | Modified-release tablet |
| 1.46254E+15 | 3.59208E+16 | Tramadol hydrochloride | 200 mg | Modified-release tablet |
| 1.46284E+15 | 3.59207E+16 | Tramadol hydrochloride | 200 mg | Modified-release capsule |
| 1.46244E+15 | 3.59206E+16 | Tramadol hydrochloride | 150 mg | Modified-release tablet |
| 1.46274E+15 | 3.59205E+16 | Tramadol hydrochloride | 150 mg | Modified-release capsule |
| 6.38914E+15 | 1.92004E+16 | Tramadol hydrochloride | 100 mg/1 ml | Oral drops |
| 1.46234E+15 | 3.59203E+16 | Tramadol hydrochloride | 100 mg | Modified-release tablet |
| 1.46264E+15 | 3.59202E+16 | Tramadol hydrochloride | 100 mg | Modified-release capsule |
| 1.70214E+15 | 322646003 | Tramadol hydrochloride | 100 mg | Effervescent powder |
| 3.99664E+15 | 1.1055E+16 | Tramadol hydrochloride | 300 mg | Modified-release tablet |
| 3.99654E+15 | 1.10548E+16 | Tramadol hydrochloride | 200 mg | Modified-release tablet |
| 3.99644E+15 | 1.10546E+16 | Tramadol hydrochloride | 100 mg | Modified-release tablet |
| 3.33284E+15 | 9.50851E+15 | Fentanyl | 75 microgram/1 hour | Transdermal patch |
| 3.33294E+15 | 9.50821E+15 | Fentanyl | 50 microgram/1 hour | Transdermal patch |
| 3.33304E+15 | 9.50801E+15 | Fentanyl | 25 microgram/1 hour | Transdermal patch |
| 3.33314E+15 | 9.50891E+15 | Fentanyl | 100 microgram/1 hour | Transdermal patch |
| 1.23502E+16 | 2.16951E+16 | Tramadol hydrochloride | 200 mg | Modified-release tablet |
| 1.23501E+16 | 2.16945E+16 | Tramadol hydrochloride | 150 mg | Modified-release tablet |
| 1.235E+16 | 2.16929E+16 | Tramadol hydrochloride | 100 mg | Modified-release tablet |
| 6.52774E+15 | 1.99574E+16 | Buprenorphine hydrochloride | 400 microgram | Sublingual tablet |
| 6.52764E+15 | 1.99572E+16 | Buprenorphine hydrochloride | 200 microgram | Sublingual tablet |
| 1.42864E+15 | 7.62111E+14 | Buprenorphine hydrochloride | 400 microgram | Sublingual tablet |
| 1.42614E+15 | 8.67611E+14 | Buprenorphine hydrochloride | 200 microgram | Sublingual tablet |
| 5.23444E+15 | 1.58509E+16 | Naloxone hydrochloride/ Oxycodone hydrochloride | 2.5 mg + 5 mg | Modified-release tablet |
| 5.23454E+15 | 1.58513E+16 | Naloxone hydrochloride/ Oxycodone hydrochloride | 20 mg + 40 mg | Modified-release tablet |
| 4.89834E+15 | 1.4976E+16 | Naloxone hydrochloride/ Oxycodone hydrochloride | 10 mg + 20 mg | Modified-release tablet |
| 4.89844E+15 | 1.49757E+16 | Naloxone hydrochloride/ Oxycodone hydrochloride | 5 mg + 10 mg | Modified-release tablet |
| 6.13214E+15 | 442341005 | Tapentadol hydrochloride | 75 mg | Tablet |
| 6.13204E+15 | 442472008 | Tapentadol hydrochloride | 50 mg | Tablet |
| 6.13314E+15 | 1.86722E+16 | Tapentadol hydrochloride | 50 mg | Modified-release tablet |
| 6.13354E+15 | 1.86721E+16 | Tapentadol hydrochloride | 250 mg | Modified-release tablet |
| 9.16014E+15 | 2.44088E+16 | Tapentadol hydrochloride | 20 mg/1 ml | Oral solution |
| 6.13344E+15 | 1.8672E+16 | Tapentadol hydrochloride | 200 mg | Modified-release tablet |
| 6.13334E+15 | 1.86719E+16 | Tapentadol hydrochloride | 150 mg | Modified-release tablet |
| 6.13324E+15 | 1.86718E+16 | Tapentadol hydrochloride | 100 mg | Modified-release tablet |
| 1.83554E+15 | 2.92611E+14 | Buprenorphine hydrochloride | 400 microgram | Sublingual tablet |
| 1.35254E+15 | 1.0411E+13 | Codeine phosphate/ Paracetamol | 30 mg + 500 mg | Effervescent tablet |
| 1.69804E+15 | 2.47311E+14 | Codeine phosphate/ Paracetamol | 30 mg + 500 mg | Capsule |
| 1.36384E+15 | 3.41211E+14 | Codeine phosphate/ Paracetamol | 30 mg + 500 mg | Tablet |
| 9.29294E+15 | 6.04311E+14 | Codeine phosphate/ Paracetamol | 12.8mg + 500 mg | Tablet |
| 1.2187E+16 | 3.40276E+16 | Oxycodone hydrochloride | 1 mg/1 ml | Oral solution |
| 9.06154E+15 | 2.36578E+16 | Oxycodone hydrochloride | 5 mg | Capsule |
| 9.06174E+15 | 2.36582E+16 | Oxycodone hydrochloride | 20 mg | Capsule |
| 1.21871E+16 | 3.40278E+16 | Oxycodone hydrochloride | 10 mg/1 ml | Oral solution |
| 9.06164E+15 | 2.3658E+16 | Oxycodone hydrochloride | 10 mg | Capsule |
| 1.27664E+15 | 3.07931E+15 | Morphine sulfate | 50 mg | Tablet |
| 1.75204E+15 | 3.45141E+15 | Morphine sulfate | 20 mg/1 ml | Oral solution |
| 1.27864E+15 | 3.07731E+15 | Morphine sulfate | 20 mg | Tablet |
| 1.75254E+15 | 3.16411E+15 | Morphine sulfate | 2 mg/1 ml | Oral solution |
| 1.27854E+15 | 2.89861E+15 | Morphine sulfate | 10 mg | Tablet |
| 1.17562E+16 | 3.34807E+16 | Buprenorphine | 5 microgram/1 hour | Transdermal patch |
| 1.17564E+16 | 3.34811E+16 | Buprenorphine | 20 microgram/1 hour | Transdermal patch |
| 1.17563E+16 | 3.34809E+16 | Buprenorphine | 10 microgram/1 hour | Transdermal patch |
| 1.26374E+16 | 3.55433E+16 | Oxycodone hydrochloride | 60 mg | Modified-release tablet |
| 1.26364E+16 | 3.55419E+16 | Oxycodone hydrochloride | 5 mg | Modified-release tablet |
| 1.26373E+16 | 3.55429E+16 | Oxycodone hydrochloride | 40 mg | Modified-release tablet |
| 1.26372E+16 | 3.55427E+16 | Oxycodone hydrochloride | 30 mg | Modified-release tablet |
| 1.26369E+16 | 3.55425E+16 | Oxycodone hydrochloride | 20 mg | Modified-release tablet |
| 1.26367E+16 | 3.55423E+16 | Oxycodone hydrochloride | 15 mg | Modified-release tablet |
| 1.26365E+16 | 3.55421E+16 | Oxycodone hydrochloride | 10 mg | Modified-release tablet |
| 1.16454E+15 | 6.80111E+14 | Dihydrocodeine tartrate/ Paracetamol | 20 mg + 500 mg | Tablet |
| 1.15754E+15 | 4.19911E+14 | Dihydrocodeine tartrate/ Paracetamol | 30 mg + 500 mg | Tablet |
| 9.17684E+15 | 2.44679E+16 | Oxycodone hydrochloride | 80 mg | Modified-release tablet |
| 9.80984E+15 | 2.79933E+16 | Oxycodone hydrochloride | 60 mg | Modified-release tablet |
| 9.17704E+15 | 2.44669E+16 | Oxycodone hydrochloride | 5 mg | Modified-release tablet |
| 9.17714E+15 | 2.44677E+16 | Oxycodone hydrochloride | 40 mg | Modified-release tablet |
| 9.80974E+15 | 2.7993E+16 | Oxycodone hydrochloride | 30 mg | Modified-release tablet |
| 9.17724E+15 | 2.44674E+16 | Oxycodone hydrochloride | 20 mg | Modified-release tablet |
| 9.80964E+15 | 2.79928E+16 | Oxycodone hydrochloride | 15 mg | Modified-release tablet |
| 9.17734E+15 | 2.44671E+16 | Oxycodone hydrochloride | 10 mg | Modified-release tablet |
| 1.21951E+16 | 3.41721E+16 | Buprenorphine | 70 microgram/1 hour | Transdermal patch |
| 1.2195E+16 | 3.41723E+16 | Buprenorphine | 52.5 microgram/1 hour | Transdermal patch |
| 1.21949E+16 | 3.41725E+16 | Buprenorphine | 35 microgram/1 hour | Transdermal patch |
| 1.17325E+16 | 3.30387E+16 | Buprenorphine | 5 microgram/1 hour | Transdermal patch |
| 1.17324E+16 | 3.30393E+16 | Buprenorphine | 20 microgram/1 hour | Transdermal patch |
| 1.17323E+16 | 3.30391E+16 | Buprenorphine | 15 microgram/1hour | Transdermal patch |
| 1.17322E+16 | 3.30389E+16 | Buprenorphine | 10 microgram/1hour | Transdermal patch |
| 1.37513E+16 | 3.87287E+16 | Buprenorphine | 5 microgram/1hour | Transdermal patch |
| 1.37515E+16 | 3.8746E+16 | Buprenorphine | 20 microgram/ 1hour | Transdermal patch |
| 1.37514E+16 | 3.87456E+16 | Buprenorphine | 10 microgram/1hour | Transdermal patch |
| 1.15772E+16 | 3.26437E+16 | Buprenorphine | 70 microgram/1hour | Transdermal patch |
| 1.15771E+16 | 3.26432E+16 | Buprenorphine | 52.5 microgram/1hour | Transdermal patch |
| 1.15079E+16 | 3.25762E+16 | Buprenorphine | 35 microgram/1 hour | Transdermal patch |
| 6.52744E+15 | 1.99566E+16 | Buprenorphine hydrochloride | 400 microgram | Sublingual tablet |
| 1.37472E+16 | 3.89564E+16 | Pethidine hydrochloride | 50 mg | Tablet |
| 1.06564E+15 | 322612004 | Pethidine hydrochloride | 50 mg | Tablet |
| 1.33006E+16 | 1.23034E+16 | Pethidine hydrochloride | 50 mg | Capsule |
| 1.04464E+15 | 322600003 | Pentazocine hydrochloride | 50 mg | Capsule |
| 1.06804E+15 | 322601004 | Pentazocine hydrochloride | 25 mg | Tablet |
| 1.04304E+15 | 4.65611E+15 | Dihydrocodeine tartrate/ Paracetamol | 7.4 mg + 500mg | Tablet |
| 1.03014E+15 | 6.55311E+14 | Codeine phosphate/ Paracetamol | 8 mg + 500 mg | Effervescent tablet |
| 4.43204E+15 | 7.72811E+14 | Codeine phosphate/ Paracetamol | 8 mg + 500 mg | Capsule |
| 1.17309E+16 | 3.30542E+16 | Buprenorphine | 5 microgram/1 hour | Transdermal patch |
| 1.17308E+16 | 3.30546E+16 | Buprenorphine | 20 microgram/1 hour | Transdermal patch |
| 1.17307E+16 | 3.30544E+16 | Buprenorphine | 10 microgram/1 hour | Transdermal patch |
| 2.96884E+15 | 8.41911E+14 | Codeine phosphate/ Paracetamol | 12.8 mg + 500 mg | Tablet |
| 1.03704E+15 | 3.87041E+15 | Hydromorphone hydrochloride | 8 mg | Modified-release capsule |
| 1.03694E+15 | 3.83851E+15 | Hydromorphone hydrochloride | 4 mg | Modified-release capsule |
| 1.03674E+15 | 3.86941E+15 | Hydromorphone hydrochloride | 2 mg | Modified-release capsule |
| 1.03684E+15 | 4.00411E+15 | Hydromorphone hydrochloride | 24 mg | Modified-release capsule |
| 1.03664E+15 | 4.00141E+15 | Hydromorphone hydrochloride | 16 mg | Modified-release capsule |
| 1.02924E+15 | 3.83751E+15 | Hydromorphone hydrochloride | 2.6 mg | Capsule |
| 1.02914E+15 | 3.83641E+15 | Hydromorphone hydrochloride | 1.3 mg | Capsule |
| 6.13384E+15 | 1.86635E+16 | Tapentadol hydrochloride | 50 mg | Modified-release tablet |
| 6.13424E+15 | 1.86652E+16 | Tapentadol hydrochloride | 250 mg | Modified-release tablet |
| 6.13414E+15 | 1.86647E+16 | Tapentadol hydrochloride | 200 mg | Modified-release tablet |
| 6.13404E+15 | 1.86644E+16 | Tapentadol hydrochloride | 150 mg | Modified-release tablet |
| 6.13394E+15 | 1.86641E+16 | Tapentadol hydrochloride | 100 mg | Modified-release tablet |
| 6.13234E+15 | 1.86629E+16 | Tapentadol hydrochloride | 75 mg | Tablet |
| 6.13224E+15 | 1.86625E+16 | Tapentadol hydrochloride | 50 mg | Tablet |
| 9.16024E+15 | 2.41208E+16 | Tapentadol hydrochloride | 20 mg/1 ml | Oral solution |
| 1.26652E+16 | 3.58474E+16 | Oxycodone hydrochloride | 80 mg | Modified-release tablet |
| 1.26651E+16 | 3.58472E+16 | Oxycodone hydrochloride | 60 mg | Modified-release tablet |
| 1.2665E+16 | 3.5846E+16 | Oxycodone hydrochloride | 5 mg | Modified-release tablet |
| 1.26649E+16 | 3.5847E+16 | Oxycodone hydrochloride | 40 mg | Modified-release tablet |
| 1.26648E+16 | 3.58468E+16 | Oxycodone hydrochloride | 30 mg | Modified-release tablet |
| 1.26647E+16 | 3.58466E+16 | Oxycodone hydrochloride | 20 mg | Modified-release tablet |
| 1.26646E+16 | 3.58464E+16 | Oxycodone hydrochloride | 15 mg | Modified-release tablet |
| 1.26645E+16 | 3.58462E+16 | Oxycodone hydrochloride | 10 mg | Modified-release tablet |
| 1.98844E+15 | 2.89821E+15 | Oxycodone hydrochloride | 1 mg/1 ml | Oral solution |
| 1.98804E+15 | 2.89571E+15 | Oxycodone hydrochloride | 5 mg | Capsule |
| 1.98824E+15 | 2.89631E+15 | Oxycodone hydrochloride | 20 mg | Capsule |
| 1.98834E+15 | 2.89751E+15 | Oxycodone hydrochloride | 10 mg/1.000ml | Oral solution |
| 1.98814E+15 | 2.89601E+15 | Oxycodone hydrochloride | 10 mg | Capsule |
| 8.04844E+15 | 2.09694E+16 | Oxycodone hydrochloride | 80 mg | Modified-release tablet |
| 8.04804E+15 | 2.09686E+16 | Oxycodone hydrochloride | 5 mg | Modified-release tablet |
| 8.04834E+15 | 2.09692E+16 | Oxycodone hydrochloride | 40 mg | Modified-release tablet |
| 8.04824E+15 | 2.0969E+16 | Oxycodone hydrochloride | 20 mg | Modified-release tablet |
| 8.04814E+15 | 2.09688E+16 | Oxycodone hydrochloride | 10 mg | Modified-release tablet |
| 1.98794E+15 | 2.89811E+15 | Oxycodone hydrochloride | 80 mg | Modified-release tablet |
| 6.12564E+15 | 1.86433E+16 | Oxycodone hydrochloride | 60 mg | Modified-release tablet |
| 2.74854E+15 | 4.07491E+15 | Oxycodone hydrochloride | 5 mg | Modified-release tablet |
| 1.98784E+15 | 2.89721E+15 | Oxycodone hydrochloride | 40 mg | Modified-release tablet |
| 6.12554E+15 | 1.86446E+16 | Oxycodone hydrochloride | 30 mg | Modified-release tablet |
| 1.98774E+15 | 2.89661E+15 | Oxycodone hydrochloride | 20 mg | Modified-release tablet |
| 6.12544E+15 | 1.86451E+16 | Oxycodone hydrochloride | 15 mg | Modified-release tablet |
| 6.12574E+15 | 1.86417E+16 | Oxycodone hydrochloride | 120 mg | Modified-release tablet |
| 1.98764E+15 | 2.89171E+15 | Oxycodone hydrochloride | 10 mg | Modified-release tablet |
| 1.98254E+15 | 3.61315E+16 | Oxycodone hydrochloride | 80 mg | Modified-release tablet |
| 6.12524E+15 | 3.61313E+16 | Oxycodone hydrochloride | 60 mg | Modified-release tablet |
| 1.98744E+15 | 3.61312E+16 | Oxycodone hydrochloride | 1 mg/1 ml | Oral solution |
| 1.37084E+16 | 3.87524E+16 | Oxycodone hydrochloride | 5 mg | Tablet |
| 2.74844E+15 | 3.6131E+16 | Oxycodone hydrochloride | 5 mg | Modified-release tablet |
| 1.98714E+15 | 322691007 | Oxycodone hydrochloride | 5 mg | Capsule |
| 1.98214E+15 | 3.61309E+16 | Oxycodone hydrochloride | 40 mg | Modified-release tablet |
| 6.12514E+15 | 3.61307E+16 | Oxycodone hydrochloride | 30 mg | Modified-release tablet |
| 1.37086E+16 | 3.87523E+16 | Oxycodone hydrochloride | 20 mg | Tablet |
| 1.98204E+15 | 3.61304E+16 | Oxycodone hydrochloride | 20 mg | Modified-release tablet |
| 1.98734E+15 | 322693005 | Oxycodone hydrochloride | 20 mg | Capsule |
| 6.12504E+15 | 3.61302E+16 | Oxycodone hydrochloride | 15 mg | Modified-release tablet |
| 6.12534E+15 | 1.86455E+16 | Oxycodone hydrochloride | 120 mg | Modified-release tablet |
| 1.98754E+15 | 3.613E+16 | Oxycodone hydrochloride | 10 mg/1 ml | Oral solution |
| 1.37085E+16 | 3.87522E+16 | Oxycodone hydrochloride | 10 mg | Tablet |
| 1.98194E+15 | 3.61295E+16 | Oxycodone hydrochloride | 10 mg | Modified-release tablet |
| 1.98724E+15 | 322692000 | Oxycodone hydrochloride | 10 mg | Capsule |
| 1.37087E+16 | 3.84567E+16 | Oxycodone hydrochloride | 5 mg | Tablet |
| 1.37089E+16 | 3.84559E+16 | Oxycodone hydrochloride | 20 mg | Tablet |
| 1.37088E+16 | 3.84533E+16 | Oxycodone hydrochloride | 10 mg | Tablet |
| 1.03338E+16 | 2.96767E+16 | Oxycodone hydrochloride | 80 mg | Modified-release tablet |
| 1.03337E+16 | 2.96774E+16 | Oxycodone hydrochloride | 60 mg | Modified-release tablet |
| 1.03331E+16 | 2.96503E+16 | Oxycodone hydrochloride | 5 mg | Modified-release tablet |
| 1.03336E+16 | 2.96776E+16 | Oxycodone hydrochloride | 40 mg | Modified-release tablet |
| 1.03335E+16 | 2.96772E+16 | Oxycodone hydrochloride | 30 mg | Modified-release tablet |
| 1.03334E+16 | 2.96783E+16 | Oxycodone hydrochloride | 20 mg | Modified-release tablet |
| 1.03333E+16 | 2.96781E+16 | Oxycodone hydrochloride | 15 mg | Modified-release tablet |
| 1.03332E+16 | 2.96778E+16 | Oxycodone hydrochloride | 10 mg | Modified-release tablet |
| 4.95624E+15 | 1.53028E+16 | Fentanyl | 75 microgram/1 hour | Transdermal patch |
| 4.95614E+15 | 1.53026E+16 | Fentanyl | 50 microgram/1 hour | Transdermal patch |
| 4.95604E+15 | 1.53024E+16 | Fentanyl | 25 microgram/1 hour | Transdermal patch |
| 5.30044E+15 | 1.60889E+16 | Fentanyl | 12 microgram/1 hour | Transdermal patch |
| 4.95634E+15 | 1.5303E+16 | Fentanyl | 100 microgram/1 hour | Transdermal patch |
| 4.50304E+15 | 1.35679E+16 | Fentanyl | 75 microgram/1 hour | Transdermal patch |
| 4.50294E+15 | 1.35677E+16 | Fentanyl | 50 microgram/1 hour | Transdermal patch |
| 4.50284E+15 | 1.35675E+16 | Fentanyl | 25 microgram/1 hour | Transdermal patch |
| 4.50314E+15 | 1.35681E+16 | Fentanyl | 100 microgram/1 hour | Transdermal patch |
| 1.01464E+15 | 3.60971E+15 | Morphine sulfate | 6 mg/1 ml | Oral solution |
| 1.01494E+15 | 3.45351E+15 | Morphine sulfate | 20 mg/1 ml | Oral solution |
| 1.01444E+15 | 3.33161E+15 | Morphine sulfate | 2 mg/1 ml | Oral solution |
| 1.01484E+15 | 3.16431E+15 | Morphine sulfate | 2 mg/1 ml | Oral solution |
| 1.01454E+15 | 3.60841E+15 | Morphine sulfate | 20 mg/1 ml | Oral solution |
| 8.88474E+15 | 3.79754E+16 | Fentanyl | 75 microgram/1 hour | Transdermal patch |
| 8.88464E+15 | 3.79749E+16 | Fentanyl | 50 microgram/1 hour | Transdermal patch |
| 8.88454E+15 | 3.79739E+16 | Fentanyl | 25 microgram/1 hour | Transdermal patch |
| 8.88444E+15 | 3.79726E+16 | Fentanyl | 12 microgram/1 hour | Transdermal patch |
| 8.88434E+15 | 3.79757E+16 | Fentanyl | 100 microgram/1 hour | Transdermal patch |
| 1.21857E+16 | 3.40522E+16 | Oxycodone hydrochloride | 80 mg | Modified-release tablet |
| 1.21856E+16 | 3.40513E+16 | Oxycodone hydrochloride | 40 mg | Modified-release tablet |
| 1.21855E+16 | 3.40519E+16 | Oxycodone hydrochloride | 20 mg | Modified-release tablet |
| 1.21853E+16 | 3.40516E+16 | Oxycodone hydrochloride | 10 mg | Modified-release tablet |
| 1.23471E+16 | 1.49836E+16 | Tramadol hydrochloride | 200 mg | Modified-release tablet |
| 1.2347E+16 | 1.49834E+16 | Tramadol hydrochloride | 150 mg | Modified-release tablet |
| 1.23469E+16 | 1.49832E+16 | Tramadol hydrochloride | 100 mg | Modified-release tablet |
| 4.42424E+15 | 1.29484E+16 | Tramadol hydrochloride | 100 mg | Modified-release tablet |
| 9.20454E+15 | 2.45608E+16 | Fentanyl | 75 microgram/1 hour | Transdermal patch |
| 9.20444E+15 | 2.45606E+16 | Fentanyl | 50 microgram/1 hour | Transdermal patch |
| 1.03368E+16 | 2.45604E+16 | Fentanyl | 25 microgram/1 hour | Transdermal patch |
| 9.20434E+15 | 2.45602E+16 | Fentanyl | 12 microgram/1 hour | Transdermal patch |
| 9.20464E+15 | 2.4561E+16 | Fentanyl | 100 microgram/1 hour | Transdermal patch |
| 9.44541E+14 | 3.88261E+15 | Morphine sulfate | 90 mg | Modified-release capsule |
| 9.44441E+14 | 3.77821E+15 | Morphine sulfate | 60 mg | Modified-release capsule |
| 9.44341E+14 | 3.65181E+15 | Morphine sulfate | 30 mg | Modified-release capsule |
| 9.44241E+14 | 4.38801E+15 | Morphine sulfate | 200 mg | Modified-release capsule |
| 9.44141E+14 | 3.88291E+15 | Morphine sulfate | 150 mg | Modified-release capsule |
| 9.44041E+14 | 3.88321E+15 | Morphine sulfate | 120 mg | Modified-release capsule |
| 9.40341E+14 | 4.08831E+15 | Morphine sulfate | 60 mg | Modified-release granules |
| 9.40841E+14 | 4.38001E+15 | Morphine sulfate | 30 mg | Modified-release granules |
| 9.40741E+14 | 4.37931E+15 | Morphine sulfate | 20 mg | Modified-release granules |
| 9.40241E+14 | 4.08921E+15 | Morphine sulfate | 200 mg | Modified-release granules |
| 9.40141E+14 | 4.08861E+15 | Morphine sulfate | 100 mg | Modified-release granules |
| 9.41241E+14 | 2.88321E+15 | Morphine sulfate | 60 mg | Modified-release tablet |
| 9.40441E+14 | 9.22411E+14 | Morphine sulfate | 5 mg | Modified-release tablet |
| 9.41141E+14 | 3.07871E+15 | Morphine sulfate | 30 mg | Modified-release tablet |
| 9.41341E+14 | 3.94011E+14 | Morphine sulfate | 200 mg | Modified-release tablet |
| 9.40041E+14 | 2.72811E+14 | Morphine sulfate | 15 mg | Modified-release tablet |
| 9.40941E+14 | 3.07771E+15 | Morphine sulfate | 10 mg | Modified-release tablet |
| 9.41041E+14 | 2.88361E+15 | Morphine sulfate | 100 mg | Modified-release tablet |
| 4.43424E+15 | 1.21437E+16 | Morphine sulfate | 1 mg/1 ml | Oral solution |
| 1.37548E+16 | 1.23005E+16 | Morphine sulfate | 100 microgram/1 ml | Oral solution |
| 9.31941E+14 | 3.63151E+15 | Morphine sulfate | 6 mg/1 ml | Oral solution |
| 9.33941E+14 | 322455007 | Morphine sulfate | 30 mg | Suppository |
| 9.31641E+14 | 3.61286E+16 | Morphine sulfate | 20 mg/1 ml | Oral solution |
| 9.34241E+14 | 322433004 | Morphine sulfate | 20 mg | Suppository |
| 9.33841E+14 | 322428003 | Morphine sulfate | 15 mg | Suppository |
| 9.31741E+14 | 3.52131E+15 | Morphine sulfate | 2 mg/1 ml | Oral solution |
| 9.31541E+14 | 3.61283E+16 | Morphine sulfate | 2 mg/1 ml | Oral solution |
| 9.34141E+14 | 322432009 | Morphine sulfate | 10 mg | Suppository |
| 9.31841E+14 | 3.63141E+15 | Morphine sulfate | 20 mg/1 ml | Oral solution |
| 9.34641E+14 | 322446009 | Morphine hydrochloride | 15 mg | Suppository |
| 7.85994E+15 | 1.21407E+16 | Morphine hydrochloride | 2 mg/1 ml | Oral solution |
| 2.06844E+15 | 3.61278E+16 | Morphine sulfate | 90 mg | Modified-release capsule |
| 9.36741E+14 | 3.61277E+16 | Morphine sulfate | 60 mg | Modified-release tablet |
| 9.30241E+14 | 4.11091E+15 | Morphine sulfate | 60 mg | Modified-release granules |
| 1.92414E+15 | 3.61276E+16 | Morphine sulfate | 60 mg | Modified-release capsule |
| 9.30341E+14 | 3.61275E+16 | Morphine sulfate | 5 mg | Modified-release tablet |
| 9.38241E+14 | 322728004 | Morphine sulfate | 50 mg | Tablet |
| 2.06884E+15 | 3.61274E+16 | Morphine sulfate | 50 mg | Modified-release capsule |
| 9.36641E+14 | 3.61273E+16 | Morphine sulfate | 30 mg | Modified-release tablet |
| 3.03294E+15 | 4.38951E+15 | Morphine sulfate | 30 mg | Modified-release granules |
| 1.92404E+15 | 3.61272E+16 | Morphine sulfate | 30 mg | Modified-release capsule |
| 9.39541E+14 | 322709006 | Morphine sulfate | 20 mg | Tablet |
| 3.03284E+15 | 4.38941E+15 | Morphine sulfate | 20 mg | Modified-release granules |
| 2.06874E+15 | 3.61271E+16 | Morphine sulfate | 20 mg | Modified-release capsule |
| 9.33141E+14 | 3.6127E+16 | Morphine sulfate | 200 mg | Modified-release tablet |
| 9.30141E+14 | 4.11081E+15 | Morphine sulfate | 200 mg | Modified-release granules |
| 1.92434E+15 | 3.61269E+16 | Morphine sulfate | 200 mg | Modified-release capsule |
| 9.29941E+14 | 3.61268E+16 | Morphine sulfate | 15 mg | Modified-release tablet |
| 2.06864E+15 | 3.61267E+16 | Morphine sulfate | 150 mg | Modified-release capsule |
| 2.06854E+15 | 3.61266E+16 | Morphine sulfate | 120 mg | Modified-release capsule |
| 9.36441E+14 | 322708003 | Morphine sulfate | 10 mg | Tablet |
| 9.29741E+14 | 3.61265E+16 | Morphine sulfate | 10 mg | Modified-release tablet |
| 1.92394E+15 | 3.61264E+16 | Morphine sulfate | 10 mg | Modified-release capsule |
| 9.36541E+14 | 3.61262E+16 | Morphine sulfate | 100 mg | Modified-release tablet |
| 9.30041E+14 | 4.11071E+15 | Morphine sulfate | 100 mg | Modified-release granules |
| 1.92424E+15 | 3.61258E+16 | Morphine sulfate | 100 mg | Modified-release capsule |
| 1.31187E+16 | 3.60226E+16 | Morphine anhydrous | 10 mg/1 ml | Oral drops/ Oral solution |
| 2.91234E+15 | 4.52721E+15 | Morphine sulfate | 60 mg | Modified-release tablet |
| 2.91224E+15 | 4.52681E+15 | Morphine sulfate | 30 mg | Modified-release tablet |
| 2.91214E+15 | 4.52591E+15 | Morphine sulfate | 10 mg | Modified-release tablet |
| 2.91244E+15 | 4.52791E+15 | Morphine sulfate | 100 mg | Modified-release tablet |
| 9.30941E+14 | 4.03501E+15 | Morphine sulfate | 50 mg | Modified-release capsule |
| 9.30841E+14 | 3.88171E+15 | Morphine sulfate | 20 mg | Modified-release capsule |
| 9.30741E+14 | 3.88221E+15 | Morphine sulfate | 100 mg | Modified-release capsule |
| 4.38684E+15 | 1.28824E+16 | Fentanyl | 75 microgram/1 hour | Transdermal patch |
| 4.38674E+15 | 1.2882E+16 | Fentanyl | 50 microgram/1 hour | Transdermal patch |
| 8.96234E+15 | 2.36823E+16 | Fentanyl | 37.5 microgram/1 hour | Transdermal patch |
| 4.38704E+15 | 1.28817E+16 | Fentanyl | 25 microgram/1 hour | Transdermal patch |
| 4.38664E+15 | 1.28815E+16 | Fentanyl | 12 microgram/1 hour | Transdermal patch |
| 4.38694E+15 | 1.28826E+16 | Fentanyl | 100 microgram/1 hour | Transdermal patch |
| 8.96441E+14 | 2.34611E+14 | Meptazinol hydrochloride | 200 mg | Tablet |
| 8.96341E+14 | 333936002 | Meptazinol hydrochloride | 200 mg | Tablet |
| 3.33154E+15 | 9.52931E+15 | Codeine phosphate/ Paracetamol | 30 mg + 500 mg | Effervescent tablet |
| 3.33454E+15 | 9.8711E+13 | Codeine phosphate/ Paracetamol | 30 mg + 500 mg | Capsule |
| 4.89914E+15 | 1.49774E+16 | Tramadol hydrochloride | 50mg | Modified-release capsule |
| 4.89944E+15 | 1.49768E+16 | Tramadol hydrochloride | 200 mg | Modified-release capsule |
| 4.89934E+15 | 1.4977E+16 | Tramadol hydrochloride | 150 mg | Modified-release capsule |
| 4.89924E+15 | 1.49772E+16 | Tramadol hydrochloride | 100 mg | Modified-release capsule |
| 4.02294E+15 | 1.10857E+16 | Fentanyl | 75 microgram/1 hour | Transdermal patch |
| 4.02284E+15 | 1.10853E+16 | Fentanyl | 50 microgram/1 hour | Transdermal patch |
| 4.02274E+15 | 1.10849E+16 | Fentanyl | 25 microgram/1 hour | Transdermal patch |
| 4.02264E+15 | 1.10845E+16 | Fentanyl | 12 microgram/1 hour | Transdermal patch |
| 4.02304E+15 | 1.10859E+16 | Fentanyl | 100 microgram/1 hour | Transdermal patch |
| 4.82444E+15 | 1.96244E+16 | Tramadol hydrochloride | 200 mg | Modified-release tablet |
| 4.82434E+15 | 1.96246E+16 | Tramadol hydrochloride | 150 mg | Modified-release tablet |
| 4.82424E+15 | 1.96248E+16 | Tramadol hydrochloride | 100 mg | Modified-release tablet |
| 1.15684E+16 | 2.46375E+16 | Tramadol hydrochloride | 200 mg | Modified-release tablet |
| 1.15681E+16 | 2.46372E+16 | Tramadol hydrochloride | 150 mg | Modified-release tablet |
| 1.15678E+16 | 2.46369E+16 | Tramadol hydrochloride | 100 mg | Modified-release tablet |
| 3.90974E+15 | 2.04754E+16 | Tramadol hydrochloride | 200 mg | Modified-release tablet |
| 3.90964E+15 | 2.04752E+16 | Tramadol hydrochloride | 150 mg | Modified-release tablet |
| 3.90954E+15 | 2.0475E+16 | Tramadol hydrochloride | 100 mg | Modified-release tablet |
| 8.53734E+15 | 2.26865E+16 | Oxycodone hydrochloride | 5 mg | Capsule |
| 8.53754E+15 | 2.26869E+16 | Oxycodone hydrochloride | 20 mg | Capsule |
| 8.53744E+15 | 2.26867E+16 | Oxycodone hydrochloride | 10 mg | Capsule |
| 7.88664E+15 | 2.09383E+16 | Oxycodone hydrochloride | 80 mg | Modified-release tablet |
| 1.0492E+16 | 2.98388E+16 | Oxycodone hydrochloride | 60 mg | Modified-release tablet |
| 7.88624E+15 | 2.09375E+16 | Oxycodone hydrochloride | 5 mg | Modified-release tablet |
| 7.88654E+15 | 2.09381E+16 | Oxycodone hydrochloride | 40 mg | Modified-release tablet |
| 1.04916E+16 | 2.98386E+16 | Oxycodone hydrochloride | 30 mg | Modified-release tablet |
| 7.88644E+15 | 2.09379E+16 | Oxycodone hydrochloride | 20 mg | Modified-release tablet |
| 1.04915E+16 | 2.98384E+16 | Oxycodone hydrochloride | 15 mg | Modified-release tablet |
| 1.04922E+16 | 2.98382E+16 | Oxycodone hydrochloride | 120 mg | Modified-release tablet |
| 7.88634E+15 | 2.09377E+16 | Oxycodone hydrochloride | 10 mg | Modified-release tablet |
| 1.18086E+16 | 3.36302E+16 | Oxycodone hydrochloride | 80 mg | Modified-release tablet |
| 1.18085E+16 | 3.363E+16 | Oxycodone hydrochloride | 60 mg | Modified-release tablet |
| 1.18084E+16 | 3.3629E+16 | Oxycodone hydrochloride | 5 mg | Modified-release tablet |
| 1.18083E+16 | 3.36298E+16 | Oxycodone hydrochloride | 40 mg | Modified-release tablet |
| 1.18082E+16 | 3.36296E+16 | Oxycodone hydrochloride | 30 mg | Modified-release tablet |
| 1.18081E+16 | 3.36294E+16 | Oxycodone hydrochloride | 20 mg | Modified-release tablet |
| 1.1808E+16 | 3.36292E+16 | Oxycodone hydrochloride | 10 mg | Modified-release tablet |
| 4.02864E+15 | 9.10121E+15 | Tramadol hydrochloride | 200 mg | Modified-release tablet |
| 4.02854E+15 | 9.10101E+15 | Tramadol hydrochloride | 150 mg | Modified-release tablet |
| 4.02834E+15 | 9.10081E+15 | Tramadol hydrochloride | 100 mg | Modified-release tablet |
| 1.74124E+15 | 3.25151E+15 | Codeine phosphate/ Paracetamol | 60 mg + 1 gram | Effervescent powder |
| 1.62184E+15 | 3.25371E+15 | Codeine phosphate/ Paracetamol | 30 mg + 500 mg | Effervescent powder |
| 7.96641E+14 | 2.32711E+14 | Codeine phosphate/ Paracetamol | 30 mg + 500 mg | Tablet |
| 3.05754E+15 | 7.33601E+15 | Codeine phosphate/ Paracetamol | 30 mg + 500 mg | Effervescent tablet |
| 1.83084E+15 | 7.37111E+14 | Codeine phosphate/ Paracetamol | 30 mg + 500 mg | Capsule |
| 6.13794E+15 | 1.7572E+16 | Codeine phosphate/ Paracetamol | 15 mg + 500 mg | Tablet |
| 1.2667E+16 | 3.5859E+16 | Oxycodone hydrochloride | 80 mg | Modified-release tablet |
| 1.26669E+16 | 3.58587E+16 | Oxycodone hydrochloride | 60 mg | Modified-release tablet |
| 1.26668E+16 | 3.58535E+16 | Oxycodone hydrochloride | 5 mg | Modified-release tablet |
| 1.26667E+16 | 3.58579E+16 | Oxycodone hydrochloride | 40 mg | Modified-release tablet |
| 1.26666E+16 | 3.58574E+16 | Oxycodone hydrochloride | 30 mg | Modified-release tablet |
| 1.26665E+16 | 3.58583E+16 | Oxycodone hydrochloride | 20 mg | Modified-release tablet |
| 1.26664E+16 | 3.58578E+16 | Oxycodone hydrochloride | 15 mg | Modified-release tablet |
| 1.26663E+16 | 3.58576E+16 | Oxycodone hydrochloride | 10 mg | Modified-release tablet |
| 4.42574E+15 | 3.24635E+16 | Fentanyl | 40 microgram/1 dose | Transdermal system |
| 1.23467E+16 | 2.19646E+16 | Tramadol hydrochloride | 200 mg | Modified-release tablet |
| 1.23466E+16 | 2.19644E+16 | Tramadol hydrochloride | 150 mg | Modified-release tablet |
| 1.23464E+16 | 2.19642E+16 | Tramadol hydrochloride | 100 mg | Modified-release tablet |
| 5.30004E+15 | 1.60341E+16 | Fentanyl citrate | 50 microgram/1 dose | Spray |
| 5.30024E+15 | 1.60353E+16 | Fentanyl citrate | 200 microgram/1 dose | Spray |
| 5.30014E+15 | 1.60347E+16 | Fentanyl citrate | 100 microgram/1 dose | Spray |
| 3.22914E+15 | 3.60452E+16 | Codeine phosphate/ Ibuprofen | 20 mg + 300 mg | Modified-release tablet |
| 7.38641E+14 | 3.60577E+16 | Hydromorphone hydrochloride | 8 mg | Modified-release capsule |
| 7.38541E+14 | 3.60576E+16 | Hydromorphone hydrochloride | 4 mg | Modified-release capsule |
| 7.38341E+14 | 3.60574E+16 | Hydromorphone hydrochloride | 2 mg | Modified-release capsule |
| 7.38441E+14 | 3.60573E+16 | Hydromorphone hydrochloride | 24 mg | Modified-release capsule |
| 7.28741E+14 | 322667003 | Hydromorphone hydrochloride | 2.6mg | Capsule |
| 7.38241E+14 | 3.60572E+16 | Hydromorphone hydrochloride | 16 mg | Modified-release capsule |
| 7.28641E+14 | 322665006 | Hydromorphone hydrochloride | 1.3 mg | Capsule |
| 8.88294E+15 | 2.34466E+16 | Buprenorphine | 70 microgram/1.000hour | Transdermal patch |
| 8.88284E+15 | 2.34464E+16 | Buprenorphine | 52.5 microgram/1 hour | Transdermal patch |
| 8.88274E+15 | 2.34462E+16 | Buprenorphine | 35 microgram/1 hour | Transdermal patch |
| 6.24441E+14 | 3.44691E+15 | Codeine phosphate | 3 mg/1 ml | Oral solution |
| 1.00457E+16 | 2.84912E+16 | Buprenorphine hydrochloride | 400 microgram | Sublingual tablet |
| 6.09341E+14 | 3.79251E+15 | Pentazocine hydrochloride | 25 mg | Tablet |
| 2.75354E+15 | 9.3411E+13 | Morphine sulfate | 60 mg | Modified-release tablet |
| 2.75344E+15 | 3.07891E+15 | Morphine sulfate | 30 mg | Modified-release tablet |
| 4.81724E+15 | 1.49306E+16 | Morphine sulfate | 200 mg | Modified-release tablet |
| 2.75334E+15 | 3.07521E+15 | Morphine sulfate | 10 mg | Modified-release tablet |
| 2.75364E+15 | 2.19511E+14 | Morphine sulfate | 100 mg | Modified-release tablet |
| 5.75241E+14 | 3.61203E+16 | Fentanyl | 75 microgram/1 hour | Transdermal patch |
| 5.75141E+14 | 3.61202E+16 | Fentanyl | 50 microgram/1 hour | Transdermal patch |
| 1.14692E+16 | 421136001 | Fentanyl | 40 microgram/1 dose | Transdermal system |
| 8.96224E+15 | 2.37077E+16 | Fentanyl | 37 microgram/1 hour | Transdermal patch |
| 5.75041E+14 | 3.612E+16 | Fentanyl | 25 microgram/1 hour | Transdermal patch |
| 3.83934E+15 | 9.75231E+15 | Fentanyl | 12 microgram/1 hour | Transdermal patch |
| 5.74941E+14 | 3.61199E+16 | Fentanyl | 100 microgram/1 hour | Transdermal patch |
| 4.89314E+15 | 1.49519E+16 | Fentanyl citrate | 100 microgram | Sublingual tablet |
| 4.42614E+15 | 1.2875E+16 | Fentanyl | 75 microgram/1 hour | Transdermal patch |
| 4.42624E+15 | 1.28749E+16 | Fentanyl | 50 microgram/1 hour | Transdermal patch |
| 4.42634E+15 | 1.28748E+16 | Fentanyl | 25 microgram/1 hour | Transdermal patch |
| 4.42604E+15 | 1.28751E+16 | Fentanyl | 100 microgram/1 hour | Transdermal patch |
| 6.44104E+15 | 1.94873E+16 | Fentanyl | 75 microgram/1 hour | Transdermal patch |
| 6.44094E+15 | 1.94871E+16 | Fentanyl | 50 microgram/1 hour | Transdermal patch |
| 6.44084E+15 | 1.94869E+16 | Fentanyl | 25 microgram/1 hour | Transdermal patch |
| 6.44074E+15 | 1.94867E+16 | Fentanyl | 12 microgram/1 hour | Transdermal patch |
| 6.44114E+15 | 1.94875E+16 | Fentanyl | 100 microgram/ 1hour | Transdermal patch |
| 1.23472E+16 | 3.45772E+16 | Dihydrocodeine tartrate/ Paracetamol | 10 mg + 500 mg | Tablet |
| 7.85914E+15 | 2.04746E+16 | Dihydrocodeine tartrate/ Paracetamol | 30 mg + 500 mg | Tablet |
| 7.85904E+15 | 2.04743E+16 | Dihydrocodeine tartrate/ Paracetamol | 20 mg + 500 mg | Tablet |
| 3.24824E+15 | 9.09011E+15 | Fentanyl | 75 microgram/1 hour | Transdermal patch |
| 3.24814E+15 | 9.08991E+15 | Fentanyl | 50 microgram/1 hour | Transdermal patch |
| 3.24804E+15 | 9.08971E+15 | Fentanyl | 25 microgram/1 hour | Transdermal patch |
| 3.83944E+15 | 9.75111E+15 | Fentanyl | 12 microgram/1 hour | Transdermal patch |
| 3.24834E+15 | 9.09031E+15 | Fentanyl | 100 microgram/1 hour | Transdermal patch |
| 4.90541E+14 | 2.83671E+15 | Fentanyl | 75 microgram/1 hour | Transdermal patch |
| 4.90441E+14 | 2.83641E+15 | Fentanyl | 50 microgram/1 hour | Transdermal patch |
| 4.90341E+14 | 2.83701E+15 | Fentanyl | 25 microgram/1 hour | Transdermal patch |
| 4.90241E+14 | 2.83811E+15 | Fentanyl | 100 microgram/1 hour | Transdermal patch |
| 1.31188E+16 | 3.71229E+16 | Morphine anhydrous | 10 mg/1 ml | Oral drops/ Oral solution |
| 2.18374E+15 | 8.05511E+14 | Tramadol hydrochloride | 400 mg | Modified-release tablet |
| 2.18364E+15 | 1.7411E+13 | Tramadol hydrochloride | 300 mg | Modified-release tablet |
| 2.18354E+15 | 1.09211E+14 | Tramadol hydrochloride | 200 mg | Modified-release tablet |
| 2.18344E+15 | 8.47811E+14 | Tramadol hydrochloride | 150 mg | Modified-release tablet |
| 2.07824E+15 | 4.24611E+14 | Tramadol hydrochloride | 75 mg | Modified-release tablet |
| 2.07854E+15 | 2.78411E+14 | Tramadol hydrochloride | 200 mg | Modified-release tablet |
| 2.07844E+15 | 9.19611E+14 | Tramadol hydrochloride | 150 mg | Modified-release tablet |
| 2.07834E+15 | 2.16911E+14 | Tramadol hydrochloride | 100 mg | Modified-release tablet |
| 8.83854E+15 | 2.30473E+16 | Oxycodone hydrochloride | 5 mg | Modified-release tablet |
| 8.83874E+15 | 2.30208E+16 | Oxycodone hydrochloride | 40 mg | Modified-release tablet |
| 8.83864E+15 | 2.30206E+16 | Oxycodone hydrochloride | 20 mg | Modified-release tablet |
| 8.83884E+15 | 2.33668E+16 | Oxycodone hydrochloride | 10 mg | Modified-release tablet |
| 3.17964E+15 | 322556006 | Cyclizine hydrochloride/ Dipipanone hydrochloride | 30 mg + 10 mg | Tablet |
| 4.69541E+14 | 3.91125E+16 | Dihydrocodeine tartrate | 90 mg | Modified-release tablet |
| 4.68741E+14 | 3.91124E+16 | Dihydrocodeine tartrate | 60 mg | Modified-release tablet |
| 4.62841E+14 | 322553003 | Dihydrocodeine tartrate | 40 mg | Tablet |
| 4.68541E+14 | 322539003 | Dihydrocodeine tartrate | 30 mg | Tablet |
| 4.69441E+14 | 3.91126E+16 | Dihydrocodeine tartrate | 120 mg | Modified-release tablet |
| 1.35829E+16 | 8.45731E+15 | Dihydrocodeine tartrate | 2 mg/1 ml | Oral suspension |
| 4.42841E+14 | 3.60986E+16 | Dihydrocodeine tartrate | 2 mg/1 ml | Oral solution |
| 4.33541E+14 | 3.03751E+15 | Dihydrocodeine tartrate | 90 mg | Modified-release tablet |
| 4.33341E+14 | 3.03711E+15 | Dihydrocodeine tartrate | 60 mg | Modified-release tablet |
| 4.33441E+14 | 3.03781E+15 | Dihydrocodeine tartrate | 120 mg | Modified-release tablet |
| 4.33141E+14 | 3.03821E+15 | Dihydrocodeine tartrate | 40 mg | Tablet |
| 3.85144E+15 | 8.42771E+15 | Dihydrocodeine tartrate/ Paracetamol | 2 mg/1 ml + 100 mg/1 ml | Oral suspension |
| 5.89124E+15 | 1.3893E+16 | Dihydrocodeine tartrate/ Paracetamol | 2 mg/1 ml + 100 mg/1 ml | Oral solution |
| 2.85004E+15 | 3.80311E+15 | Codeine phosphate/ Paracetamol | 15 mg + 500 mg | Tablet |
| 6.38654E+15 | 1.92072E+16 | Codeine phosphate/ Paracetamol | 15 mg + 500 mg | Effervescent tablet |
| 6.43144E+15 | 1.91919E+16 | Codeine phosphate/ Paracetamol | 15 mg + 500 mg | Capsule |
| 3.71341E+14 | 322504003 | Codeine phosphate | 60 mg | Tablet |
| 3.71241E+14 | 322503009 | Codeine phosphate | 30 mg | Tablet |
| 3.71141E+14 | 322502004 | Codeine phosphate | 15 mg | Tablet |
| 3.36741E+14 | 3.42011E+15 | Codeine phosphate/ Ibuprofen | 20 mg + 300 mg | Modified-release tablet |
| 3.72941E+14 | 322307006 | Codeine phosphate/ Paracetamol | 8 mg + 500 mg | Tablet |
| 1.34975E+16 | 3.85552E+16 | Codeine phosphate/ Paracetamol | 8 mg + 500 mg | Effervescent tablet |
| 3.73041E+14 | 322343000 | Codeine phosphate/ Paracetamol | 8 mg + 500 mg | Effervescent tablet |
| 2.94841E+14 | 322344006 | Codeine phosphate/ Paracetamol | 8 mg + 500 mg | Capsule |
| 4.59064E+15 | 1.15792E+16 | Codeine phosphate/ Paracetamol | 8 mg + 500 mg | Tablet |
| 1.23561E+16 | 3.46253E+16 | Codeine phosphate/ Paracetamol | 60mg + 1 gram | Tablet |
| 1.34177E+16 | 3.80639E+16 | Codeine phosphate/ Paracetamol | 6 mg/1 ml + 100 mg/1 ml | Oral solution |
| 3.70641E+14 | 322341003 | Codeine phosphate/ Paracetamol | 30 mg + 500 mg | Tablet |
| 3.26141E+14 | 322365000 | Codeine phosphate/ Paracetamol | 30 mg + 500 mg | Effervescent tablet |
| 1.58874E+15 | 322323006 | Codeine phosphate/ Paracetamol | 30 mg + 500 mg | Effervescent powder |
| 2.95441E+14 | 322366004 | Codeine phosphate/ Paracetamol | 30 mg + 500 mg | Capsule |
| 2.87534E+15 | 3.80561E+15 | Codeine phosphate/ Paracetamol | 15 mg + 500 mg | Tablet |
| 6.38644E+15 | 1.92307E+16 | Codeine phosphate/ Paracetamol | 15 mg + 500 mg | Effervescent tablet |
| 6.43134E+15 | 1.92002E+16 | Codeine phosphate/ Paracetamol | 15 mg + 500 mg | Capsule |
| 5.33454E+15 | 322379008 | Codeine phosphate/ Paracetamol | 12.8 mg + 500 mg | Tablet |
| 1.26037E+16 | 3.55441E+16 | Buprenorphine | 70 microgram/1 hour | Transdermal patch |
| 1.26036E+16 | 3.55439E+16 | Buprenorphine | 52.5 microgram/1 hour | Transdermal patch |
| 1.26035E+16 | 3.55437E+16 | Buprenorphine | 35 microgram/1 hour | Transdermal patch |
| 1.14898E+16 | 3.21973E+16 | Oxycodone hydrochloride | 80 mg | Modified-release tablet |
| 1.09848E+16 | 3.13222E+16 | Oxycodone hydrochloride | 5 mg | Modified-release tablet |
| 1.14897E+16 | 3.21968E+16 | Oxycodone hydrochloride | 40 mg | Modified-release tablet |
| 1.09851E+16 | 3.1324E+16 | Oxycodone hydrochloride | 20 mg | Modified-release tablet |
| 1.09849E+16 | 3.13235E+16 | Oxycodone hydrochloride | 10 mg | Modified-release tablet |
| 3.34364E+15 | 9.56531E+15 | Buprenorphine | 5 microgram/1 hour | Transdermal patch |
| 3.34384E+15 | 9.56591E+15 | Buprenorphine | 20 microgram/1 hour | Transdermal patch |
| 1.10772E+16 | 3.18772E+16 | Buprenorphine | 15 microgram/1 hour | Transdermal patch |
| 3.34374E+15 | 9.56561E+15 | Buprenorphine | 10 microgram/1 hour | Transdermal patch |
| 1.10293E+16 | 3.12792E+16 | Buprenorphine | 5 microgram/1 hour | Transdermal patch |
| 1.10295E+16 | 3.12786E+16 | Buprenorphine | 20 microgram/1 hour | Transdermal patch |
| 1.21068E+16 | 3.40274E+16 | Buprenorphine | 15 microgram/1 hour | Transdermal patch |
| 1.10294E+16 | 3.12789E+16 | Buprenorphine | 10 microgram/ 1hour | Transdermal patch |
| 1.24095E+16 | 3.48388E+16 | Buprenorphine | 5 microgram/1 hour | Transdermal patch |
| 1.24097E+16 | 3.48392E+16 | Buprenorphine | 20 microgram/1 hour | Transdermal patch |
| 1.24096E+16 | 3.4839E+16 | Buprenorphine | 10 microgram/1 hour | Transdermal patch |
| 2.73774E+15 | 3.59139E+16 | Buprenorphine | 70 microgram/1 hour | Transdermal patch |
| 3.34334E+15 | 9.56721E+15 | Buprenorphine | 5 microgram/1 hour | Transdermal patch |
| 2.73764E+15 | 3.59138E+16 | Buprenorphine | 52.5microgram/1 hour | Transdermal patch |
| 1.74241E+14 | 322492007 | Buprenorphine hydrochloride | 400 microgram | Sublingual tablet |
| 2.73754E+15 | 3.59137E+16 | Buprenorphine | 35 microgram/1 hour | Transdermal patch |
| 2.92264E+15 | 3.59136E+16 | Buprenorphine hydrochloride | 300 microgram/1 ml | Solution for injection |
| 3.34354E+15 | 9.56731E+15 | Buprenorphine | 20 microgram/1 hour | Transdermal patch |
| 1.72641E+14 | 322498006 | Buprenorphine hydrochloride | 200 microgram | Sublingual tablet |
| 1.1077E+16 | 3.20384E+16 | Buprenorphine | 15 microgram/1 hour | Transdermal patch |
| 3.34344E+15 | 9.56741E+15 | Buprenorphine | 10 microgram/1 hour | Transdermal patch |
| 1.23257E+16 | 3.45518E+16 | Buprenorphine | 5 microgram/1 hour | Transdermal patch |
| 1.2326E+16 | 3.45522E+16 | Buprenorphine | 20 microgram/1 hour | Transdermal patch |
| 1.23259E+16 | 3.4552E+16 | Buprenorphine | 10 microgram/1 hour | Transdermal patch |
| 1.17808E+16 | 3.35481E+16 | Buprenorphine | 70 microgram/1 hour | Transdermal patch |
| 1.17807E+16 | 3.35464E+16 | Buprenorphine | 52.5 microgram/1 hour | Transdermal patch |
| 1.17806E+16 | 3.35466E+16 | Buprenorphine | 35 microgram/1 hour | Transdermal patch |
| 1.14845E+16 | 3.24846E+16 | Buprenorphine | 70 microgram/1 hour | Transdermal patch |
| 1.14844E+16 | 3.24844E+16 | Buprenorphine | 52.5 microgram/1 hour | Transdermal patch |
| 1.14843E+16 | 3.24842E+16 | Buprenorphine | 35 microgram/1 hour | Transdermal patch |
| 1.26034E+16 | 3.55443E+16 | Buprenorphine | 5 microgram/1 hour | Transdermal patch |
| 1.26033E+16 | 3.55451E+16 | Buprenorphine | 20 microgram/1 hour | Transdermal patch |
| 1.26032E+16 | 3.55445E+16 | Buprenorphine | 10 microgram/1 hour | Transdermal patch |
| 1.3712E+16 | 3.88113E+16 | Tramadol hydrochloride | 200 mg | Modified-release tablet |
| 1.2389E+16 | 3.47334E+16 | Tramadol hydrochloride | 100 mg | Modified-release tablet |
| 1.06421E+16 | 3.07217E+16 | Oxycodone hydrochloride | 80 mg | Modified-release tablet |
| 1.0642E+16 | 3.07214E+16 | Oxycodone hydrochloride | 60 mg | Modified-release tablet |
| 1.06414E+16 | 3.07193E+16 | Oxycodone hydrochloride | 5 mg | Modified-release tablet |
| 1.06419E+16 | 3.07208E+16 | Oxycodone hydrochloride | 40 mg | Modified-release tablet |
| 1.06418E+16 | 3.07211E+16 | Oxycodone hydrochloride | 30 mg | Modified-release tablet |
| 1.06417E+16 | 3.07205E+16 | Oxycodone hydrochloride | 20 mg | Modified-release tablet |
| 1.06416E+16 | 3.07201E+16 | Oxycodone hydrochloride | 15 mg | Modified-release tablet |
| 1.06415E+16 | 3.07198E+16 | Oxycodone hydrochloride | 10 mg | Modified-release tablet |
| 1.06314E+15 | 322604007 | Pentazocine lactate | 50 mg | Suppository |
| 3.68541E+14 | 3.65661E+16 | Codeine phosphate | 5 mg/1 ml | Oral solution |
| 2.64504E+15 | 3.88964E+16 | Codeine phosphate | 3 mg/1 ml | Oral solution |

**Table S3: Decisions made to prepare opioid prescriptions using DrugPrep algorithm**

| Step  **A.Data cleaning** | | | | | |
| --- | --- | --- | --- | --- | --- |
| Decision node:  Raw data | **1**  **Clean implausible qty** | **2**  **Clean missing qty** | **3**  **Clean implausible ndd** | **4**  **Clean missing ndd** | **5**  **Clean all available duration variables** |
|  | a. Use implausible value  b. Set to missing  c. Set to individual median  d. set to population median  e. Use previous value  f. Use next value | a. Keep as missing  b.set to individual median  c.set to population median  d.use previous value  e.use next value | a. use implausible value  b.set to missing  c. set to imdividual median  d. set to population median  e. use previous value  f. use next value | a. keep as missing  b.set to individual median  C. set to population median  d.use previous value  e. use next value | a.Do nothing  b(6). Set to missing if >6months  b(12).set to missing if >12 months  b(24). Set to missing if >24 months  c(6). Set to 6 months if >6 months  c(12). Set to 12 months if > 12 months  c. calculated for each prescription by dividing the imputed quantity by numeric daily doses. |
| Step | **B. Define prescription length** | | **C. Handle concurrent &sequential prescriptions** | | |
| Decision node: | **6**  **Generate stop dates** | **7**  **Clean missing stop dates** | **8**  **Handle multiple prescriptions** | **9**  **Handle overlapping prescriptions** | **10**  **Handle gaps between prescriptions** |
|  | a.Start + numdays  b.start+dose_duration  c.start+qty/ndd  d.(15)use mean if gap<15 days  d(30) use mean if gap<30 days  d(60) use mean if gap <60 days  d(90)use mean if gap <90 days  d. use mean regardless of gap | a.keep missing  b.set to individual mean  c.set to population mean  d.use population mean if individual mean is missing | a.do nothing  b.use mean ndd & duration  c.use prescription with smallest ndd  d.use prescription with largest ndd  e.use shortest prescription  f.use longest prescription  g.sum durations | a.Ignore overlap  b.Add overlap to end 2^nd^ prescription | a.do nothing :allow gap  b(15)assume continuous us eif gap <15b days  b(30) assume continuous use if gap <30 days  b(60)assume continuous us if gap < 60 days |

**Table S4: Equianalgesic ratios to calculate Oral Morphine Equivalent Doses**

| Opioid* | Form** | Equianalgesic ratio*** |
| --- | --- | --- |
| Buprenorphine | Transdermal patch  Sublingual tablet | 1.8  10 |
| Codeine |  | 0.15 |
| Codeine/ Ibuprofen |  | 0.15 |
| Codeine/ Paracetamol |  | 0.15 |
| Dihydrocodeine |  | 0.25 |
| Dihydrocodeine/Paracetamol |  | 0.25 |
| Fentanyl | Transdermal patch  Sublingual tablet  Nasal spray | 2.4  0.13  0.16 |
| Hydromorphone |  | 4 |
| Meptazinol |  | 0.03 |
| Morphine Sulfate |  | 1 |
| Naloxone/Oxycodone |  | 1.50 |
| Oxycodone |  | 1.50 |
| Pentazocine |  | 0.37 |
| Pethidine |  | 0.10 |
| Tapentadol |  | 0.40 |
| Tramadol |  | 0.20 |
| Cyclizine/Dipipanone |  | 0.5 |

*Opioid doses are in mg.day^-1^ except for fentanyl transdermal (in μg.h^-1^)

**Form refers to an oral preparation unless otherwise stated

***Equianalgesic ratio: the potency of respective opioid/opioid formulations compared with oral morphine.

**Table S5: Baseline characteristics of patients undergoing colectomy between the years 2010 and 2019**

| Variable | Years | | | | | | | | | |
| --- | --- | --- | --- | --- | --- | --- | --- | --- | --- | --- |
|  | **2010**  **N= 8001** | **2011**  **N=** **8470** | **2012**  **N= 8850** | **2013**  **N= 8963** | **2014**  **N=** **9038** | **2015**  **N=** **9614** | **2016**  **N= 10 009** | **2017**  **N=** **10 286** | **2018**  **N=** **10 794** | **2019**  **N=** **11 130** |
| Age, years | 66.8 | 66.9 | 66.3 | 65.8 | 66.3 | 65.2 | 65.0 | 65.0 | 63.9 | 63.9 |
| Sex  Female  Male | 3938 (49.2)  4063 (50.8) | 4087 (48.3)  4383 (51.8) | 4377 (49.5)  4473 (50.5) | 4417 (49.3)  4546 (50.7) | 4489 (49.7)  4549 (50.3) | 4853 (50.5)  4761 (49.5) | 5130 (51.3)  4879 (48.8) | 5150 (50.1)  5136 (50.1) | 5561 (51.5)  5233 (48.5) | 5776 (51.9)  5354 (48.1) |
| Preoperative opioid | | | | | | | | | | |
| Naïve  Currently  Previously | 6326 (79.1)  1404 (17.6)  271 (3.4) | 6715 (79.3)  1410 (16.7)  345 (4.1) | 7097 (80.2)  1440 (16.3)  313 (3.5) | 7242 (80.8)  1406 (15.7)  315 (3.5) | 7345 (81.3)  170 (15.2)  323 (3.6) | 7878 (81.9)  1396 (14.5)  340 (3.54) | 8307 (83.0)  1368 (13.7)  334 (3.3) | 8571 (83.3)  1393 (13.5)  322 (3.13) | 9178 (85.1)  1302 (12.1)  314 (2.9) | 9674 (86.9)  1161 (10.4)  295 (2.7) |
| Index of Multiple Deprivation | | | | | | | | | | |
| 1  2  3  4  5  Missing | 1775 (22.2)  1795 (22.4)  1677 (20.9)  1439 (17.9)  1307 (16.3)  8 (0.10) | 1934 (22.8)  1820 (21.8)  1719 (20.3)  1617 (19.1)  1370 (16.2)  10 (0.12) | 1979 (22.4)  1835 (20.7)  1842 (20.8)  1608 (18.2)  1572 (17.8)  14 (0.16) | 2015 (22.5)  1928 (21.5)  1768 (19.7)  1726 (19.3)  1519 (16.9)  7 (0.08) | 2035 (22.5)  1857 (20.6)  1810 (20.0)  1777 (19.7)  1550 (17.2)  9 (0.10) | 2154 (22.4)  2005 (20.9)  1957 (20.4)  1786 (18.6)  1703 (17.7)  9 (0.09) | 2198 (21.9)  2125 (21.2)  2063 (20.6)  1898 (18.9)  1706 (17.0)  19 (0.19) | 2328 (22.6)  2203 (21.4)  2064 (20.1)  1961 (19.1)  1710 (16.6)  20 (0.19) | 2415 (22.4)  2304 (21.4)  2099 (19.5)  2138 (19.8)  1827 (16.9)  11 (0.10) | 2459 (22.1)  2390 (21.5)  2270 (20.4)  2116 (19.0)  1883 (16.9)  12 (0.11) |
| Charlson comorbidity index | | | | | | | | | | |
| 0  1  ≥2 | 1968 (24.6)  608 (7.6)  5425 (67.8) | 1859 (21.9)  649 (7.7)  5962 (70.4) | 2090 (23.6)  668 (7.55)  6092 (68.8) | 2249 (25.1)  648 (7.2)  6066 (67.7) | 2178 (24.1)  639 (7.1)  6221 (68.8) | 2433 (25.3)  725 (7.5)  6456 (67.2) | 2412 (24.1)  772 (7.7)  6825 (68.2) | 2361 (22.9)  791 (7.7)  7134 (69.4) | 2588 (23.9)  791 (7.3)  7415 (68.7) | 2591 (23.3)  789 (7.1)  7750 (69.6) |
| Surgical approach | | | | | | | | | | |
| Open  Minimally invasive | 5812 (72.6)  2189 (27.4) | 5915 (69.8)  2555 (30.2) | 5973 (67.5)  2877 (32.5) | 5796 (64.7)  3167 (35.3) | 5606 (62.0)  3432 (37.9) | 5661 (58.9)  3953 (41.1) | 5707 (57.0)  4302 (42.9) | 5523 (53.7)  4763 (46.3) | 5661 (52.5)  5133 (47.5) | 5385 (48.4)  5745 (51.6) |
| Cancer diagnosis | | | | | | | | | | |
| No  Yes | 3367 (42.1)  4634 (57.9) | 3419 (40.4)  5051 (59.6) | 3725 (42.1)  5125 (57.9) | 3913 (43.7)  5050 (56.3) | 3947 (43.7)  5091 (56.3) | 4369 (45.4)  5245 (54.6) | 4452 (44.5)  5557 (55.5) | 4610 (44.8)  5676 (55.2) | 4858 (45.0)  5936 (54.9) | 5028 (45.2)  6102 (54.8) |
| Admission type | | | | | | | | | | |
| Elective  Emergency | 5735 (71.7)  2266 (28.3) | 5948 (70.2)  2522 (29.8) | 6229 (70.4)  2621 (29.6) | 6185 (69.0)  2778 (30.9) | 6340 (70.2)  2698 (29.9) | 6715 (69.8)  2899 (30.2) | 6963 (69.6)  3046 (30.4) | 7348 (71.4)  2938 (28.6) | 7609 (70.5)  3185 (29.5) | 7977 (71.7)  3153 (28.3) |

**Table S6: Changes in the potency of opioid prescribed in initial prescription received after discharge**

| **Stratified by Opioid exposure before colectomy** |  | | **Opioid potency** | | |
| --- | --- | --- | --- | --- | --- |
|  |  |  | **Weak opioids** | **Strong opioids** | **Both weak & strong** |
|  | **Naïve**  **N=6981** | 2010 n=721 | 87.9% | 9.0% | 3.0% |
|  |  | 2019 n=647 | 75.1% | 20.9% | 3.8% |
|  |  | Percent change, p value | -14.8%, p<0.001 | +132.9%, p<0.001 | +26.3%, p=0.436 |
|  | **Currently**  **N=7747** | 2010 n=808 | 77.4% | 17.5% | 5.0% |
|  |  | 2019 n=677 | 64.8% | 29.4% | 5.9% |
|  |  | Percent change, p value | -16.3%, p<0.001 | +67.9%, p<0.001 | +18.4%, p=0.476 |
|  | **previously**  **N=775** | 2010 n=78 | 83.7% | 6.5% | 9.8% |
|  |  | 2019 n=74 | 71.4% | 28.6% | 0% |
|  |  | Percent change, p value | 14.7%, p=0.055 | +338.7%, p<0.001 | -100%, p<0.005 |
| **Stratified by surgical approach** | **Open**  **N=10,308** | 2010 n=1326 | 80.0% | 14.9% | 4.8% |
|  |  | 2019 n=861 | 63.0% | 30.9% | 5.4% |
|  |  | Percent change, p value | -21.3 %, p<0.001 | +106.0%, p<0.001 | 12.5%, p=0.510 |
|  | **Laparoscopic**  **N= 5,195** | 2010 n=420 | 88.5% | 8.1% | 3.3% |
|  |  | 2019 n=662 | 78.1% | 18.3% | 3.6% |
|  |  | Percent change, p value | -11.8%, p<0.001 | +125.9%, p<0.001 | +9%, p=0.799 |

**N represents total number of patients in this strata, n = number of patients in this stratum in certain year.**

**Table S7: Changes in the type of opioid prescribed in initial prescription received after discharge**

**Changes in type of opioid prescribed for the overall cohort**

| Opioids analgesics | | 2010  n=1746 | 2019  n=1523 | Absolute change | Percent change | P value |
| --- | --- | --- | --- | --- | --- | --- |
| Weak Opioids | Codeine | 43.5% | 49.8% | +6.3 | +14.5% | **<0.001** |
|  | Dihydrocodeine | 4.0% | 3.2% | -0.90 | -21.5% | **0.043** |
|  | Tramadol | 36.8% | 18.9% | -17.8 | -48.4% | **<0.001** |
| Strong opioids | Morphine | 8.6% | 15.2% | +6.6 | +76.9% | **<0.001** |
|  | Oxycodone | 3.4% | 7.8 % | +4.4 | +131.1% | **<0.001** |
|  | Buprenorphine | 1.4% | 2.5% | + 1.1 | +74.8% | **0.041** |
|  | Fentanyl | 2.1% | 2.2% | + 0.10 | +2.4% | 0.920 |

**Changes in type of opioid prescribed stratified by surgical approach**

|  | **Surgical approach** | | | | | | | | | |
| --- | --- | --- | --- | --- | --- | --- | --- | --- | --- | --- |
|  | **Open colectomy** | | | | | **Laparoscopic** | | | | |
| **Opioid analgesics** | **2010**  **n=1326** | **2019**  **n=861** | **Absolute change** | **Percent change** | **P value** | **2010**  **n=420** | **2019**  **n=662** | **Absolute change** | **Percent change** | **P value** |
| **Codeine** | 43.2 | 46.9 | +3.8 | +8.9% | 0.054 | 44.5 | 53.5 | +9.0 | +20.2% | **<0.001** |
| **Dihydrocodeine** | 4.07 | 3.02 | -1.9 | -25.8% | **0.0259** | 3.8 | 3.3 | -0.49 | -12.9 | 0.822 |
| **Tramadol** | 35.3 | 15.9 | -19.4 | -54.9% | **<0.001** | 41.4 | 22.9 | -18.5 | -44.5% | 0.326 |
| **Morphine** | 9.2 | 18.3 | +9.2 | +98.9% | **<0.001** | 6.7 | 11.0 | +4.3 | +65.3% | **0.007** |
| **Oxycodone** | 4.1 | 8.6 | +4.5 | +110.9% | **<0.001** | 1.2 | 6.8 | +5.6 | +470.9 | **<0.001** |
| **Buprenorphine** | 1.5 | 3.5 | +1.9 | +130.0% | **0.003** | 1.2 | 1.2 | +0.02 | +1.7 % | 0.925 |
| **Fentanyl** | 2.4 | 2.9 | +0.49 | +20.3% | 0.317 | 1.2 | 1.2 | +0.02 | +1.7 % | **<0.001** |

**Changes in type of opioid prescribed stratified by opioid exposure before colectomy**

| **Opioid analgesics** | **Opioid exposure before surgery** | | | | | | | | | | | | | | |
| --- | --- | --- | --- | --- | --- | --- | --- | --- | --- | --- | --- | --- | --- | --- | --- |
|  | **Naïve n=7382** | | | | | **Currently exposed n=8676** | | | | | **Previously exposed n=828** | | | | |
|  | **2010**  **N=756** | **2019**  **N=686** | **Absolute change** | **Percent change** | **P value** | **2010**  **N=898** | **2019**  **N=760** | **Absolute change** | **Percent change** | **P value** | **2010**  **N=92** | **2019**  **N=77** | **Absolute** | **Percent** | **P value** |
| Codeine | 42.2% | 55.7% | +13.5 | +31.9% | **<0.001** | 43.8% | 44.5% | +0.73 | +1.6% | 0.779 | 52.1% | 50.7% | -1.4 | -2.7% | 0.552 |
| Dihydrocodeine | 3.8% | 4.2% | +0.39 | +10.1% | 0.904 | 4.5% | 2.37% | -2.1 | -46.7% | 0.019 | 1.1% | 1.3% | +0.21 | +19.3% | **0.054** |
| Tramadol | 43.1% | 16.9% | -26.2 | -60.8% | **<0.001** | 31.7% | 20.8% | -20.8 | -34.4% | <0.001 | 33.7% | 19.5% | -14.2 | -42.1% | **0.021** |
| Morphine | 7.7% | 12.5% | +4.8 | +62.9% | **<0.001** | 9.8% | 17.5% | +7.7 | +78.6% | <0.001 | 4.4% | 15.6% | +11.3 | +258.1% | 0.057 |
| Oxycodone | 1.6% | 7.8% | +6.3 | +395.3% | **<0.001** | 4.7% | 7.6% | +2.9 | +63.2% | <0.001 | 5.4% | 9.1% | +3.7 | +67.4% | 0.532 |
| Buprenorphine | 0.53% | 0.87% | +0.34 | +64.1% | 0.429 | 2.2% | 4.1% | +1.9 | +82.9% | 0.032 | 1.1% | 1.3% | +0.21 | +19.3% | 0.667 |
| Fentanyl | 0.93% | 1.5% | +0.53 | +56.9% | 0.275 | 3.2% | 2.8% | -0.47 | -14.5% | 0.607 | 1.1% | 2.6% | +1.5 | +138.1% | 0.540 |

**n represents total number of patients in this strata, N = number of patients in this stratum in certain year.**
